# Syntaxin-2 balances phagocytic uptake and phagolysosomal clearance in macrophages

**DOI:** 10.1242/jcs.263855

**Published:** 2025-08-06

**Authors:** Suman Samanta, Abhrajyoti Nandi, Rupak Datta, Subhankar Dolai

**Affiliations:** ^1^ Department of Biological Sciences, Indian Institute of Science Education and Research Kolkata, Mohanpur, West Bengal, 741246, India; ^2^ Department of Zoology, Institute of Science, Banaras Hindu University, Varanasi, 221005, India

**Keywords:** Macrophage, Stx2, SNARE, Phagocytosis, Fc receptors, Lysosome

## Abstract

Phagocytosis engulfs receptor-bound particles within phagosomes that mature into acidic, hydrolase-enriched phagolysosomes for content degradation. Although an essential process for host defense and homeostasis, defective or uncontrolled phagocytosis can be detrimental. We report here, that syntaxin-2 (Stx2), a poorly characterized SNARE in phagocytes, defines the course of macrophage phagocytosis by coordinating surface receptor density, phagosome biogenesis and maturation. Stx2 is expressed primarily on the plasma membrane, early endosomes and phagosomes. Stx2 knockdown (Stx2-KD) increases entrapment and uptake of IgG-opsonized particles owing to dysregulated formation and expansion of phagocytic cups driven by elevated IgG receptor recycling and trafficking of early endosomes and VAMP4-positive post-Golgi compartments to phagocytic cups. Interestingly, Stx2-KD decreases secretion of pro-cathepsins and increases lysosome content. However, Stx2-KD impedes phagosome maturation by preventing coalescence with late endosomes, lysosomes and reducing phagosomal acidification. Consequently, Stx2-depleted macrophages exhibit aberrant uptake of IgG-opsonized bacteria and impaired digestion, resulting in increased intracellular accumulation of intact bacteria. Collectively, Stx2 critically balances phagocytic uptake and phagolysosomal clearance in macrophages, suggesting that Stx2 could be an attractive target to modulate phagocytosis plasticity and to control aberrant phagocytosis.

## Teaser

**Summary:** Macrophage-mediated homeostatic phagocytosis becomes detrimental when dysregulated. Syntaxin-2, a SNARE protein, differentially regulates the ingestion and clearance of phagocytic particles and might be crucial in controlling aberrant phagocytosis.

## INTRODUCTION

Phagocytosis, a conserved cellular process, engulfs and eliminates large (>0.5 µm in diameter) extracellular particles (Aderem and Underhill, 1999; Flannagan et al., 2012). Although various epithelial and non-epithelial cells display phagocytic activity (Rabinovitch, 1995), professional phagocytes of myeloid origin, such as macrophages, actively eliminate transformed cells, cellular debris, pathological aggregates and invading pathogens (Flannagan et al., 2012; Kamber et al., 2021). Phagocytosis begins with the engagement of phagocytic particles with specific cell surface receptors such as Fc receptors (FcR) that binds to Fc region of opsonizing IgG (Aderem and Underhill, 1999; Flannagan et al., 2012). Activation of phagocytic receptors triggers actin polymerization that restructures the plasma membrane into cup-like configurations (phagocytic cups) around the particles (Freeman and Grinstein, 2014; Swanson, 2008). Expansion of phagocytic cups leads to sequestration of particles within vesicular phagosomes. These nascent phagosomes mature into acidic, hydrolytic enzyme-rich phagolysosomes to degrade and recycle engulfed particles (Botelho and Grinstein, 2011; Henry et al., 2004). Components of the degraded materials also cross-present as antigens to activate adaptive immunity (Mantegazza et al., 2013). Being so crucial for organismal homeostasis, disease defense and immunity, aberrant phagocytosis can be detrimental. Uncontrolled phagocytosis leads to excessive red blood cell destruction (hemophagocytosis), tissue damage and neurodegeneration (Al-Samkari and Berliner, 2018; Butler et al., 2021), whereas impaired clearance of phagocytic bodies have detrimental roles in autoimmune, immunodeficiency and neuroinflammatory diseases (Kleinberger et al., 2014; Muñoz et al., 2010). Hence, phagocytosis requires intense control over uptake and clearance to thwart adverse consequences. Machineries that promote or inhibit phagocytosis, and understanding their mechanisms, are thus extremely important (Freeman and Grinstein, 2021; Gordon et al., 2017; Pluvinage et al., 2019).

Abundance of macrophage surface receptors, expansion of phagocytic cups and maturation of phagosomes determine the extent of particle engagement, engulfment and clearance (Flannagan et al., 2012; Jaumouille and Grinstein, 2011). Regulated fusion of FcR carrying recycling compartments to plasma membrane delivers surface receptors, including FcR (Cox et al., 2000; Flannagan et al., 2012). Acquisition of membrane for phagocytic cup expansion (for phagosome biogenesis) (Flannagan et al., 2012) and subsequent maturation of phagosomes into degradative phagolysosomes also requires regulated fusion with various endolysosomal compartments (Becken et al., 2010; Flannagan et al., 2012; Vieira et al., 2002). In eukaryotes, soluble N-ethylmaleimide-sensitive factor attachment protein receptor (SNARE) proteins mediate membrane fusions (Jahn and Scheller, 2006; Sudhof and Rothman, 2009). Typically, two target membrane-localized t-SNAREs (syntaxins and SNAPs) combine with one vesicle-associated v-SNAREs (like VAMPs) to form a four-helix trans-SNARE complex (SNARE-pin) that mediates membrane merger (Jahn and Scheller, 2006; Sudhof and Rothman, 2009). SNARE-dependent trafficking of early endosomes (Hackam et al., 1998), recycling endosomes (Bajno et al., 2000; Niedergang et al., 2003), late endosomes (Braun et al., 2004) and lysosomes (Czibener et al., 2006; Rao et al., 2004) promote phagocytosis. However, machineries and mechanisms that coordinate endolysosomal trafficking to balance phagocytic uptake (phagosome biogenesis) and phagolysosomal clearance remain largely unknown.

Contrary to pro-fusion roles, some SNAREs (inhibitory- or i-SNAREs) interfere with other cis-SNAREs in forming the fusogenic SNARE-pin to fine-tune membrane fusions (Paumet et al., 2009; Varlamov et al., 2004). Syntaxin-2 (Stx2) was initially discovered as an extracellular epithelial morphogen called epimorphin (Fritsch et al., 2002; Hirai et al., 1992), and later, its intracellular version was characterized as a t-SNARE (Bennett et al., 1993). Stx2 is unique among all the known t-SNAREs in showing fusion-neutral (in platelets) (Ye et al., 2012), fusion-competent (in kidney and goblet cell) (Low et al., 2003; Wojnacki et al., 2023) and i-SNARE properties (in pancreatic acinar and β-cells) (Dolai et al., 2018; Zhu et al., 2017). Considering the various Stx2 roles in membrane fusions, coupled with prominent expression in macrophages (Hackam et al., 1996; Murray et al., 2005; Pagan et al., 2003) but unknown functions in phagocytosis, we hypothesized that Stx2 might be a crucial component that can differentially control the delivery of diverse intracellular compartments to shape the course of phagocytosis.

We found that Stx2 is indeed a crucial regulator of macrophage endomembrane trafficking, essential for controlling phagocytic uptake and phagosome maturation. Stx2 restricts recycling of receptor carrying vesicles to curtail surface FcR and IgG-opsonized particle engagement. During phagocytosis, Stx2 also inhibits acquisition of early endosomes and VAMP4-positive post-Golgi vesicles to restrain dysregulated expansion of phagocytic cups, and hence phagosome biogenesis. In contrast, Stx2 promotes phagosome maturation by the acquisition of late endosomes and lysosomes. Stx2 also facilitates macrophage secretion of pro-cathepsins to curb cellular lysosome contents. Therefore, Stx2-depleted macrophages lose control over phagocytosis and show unabated particle uptake but impaired degradation, despite having more lysosomes. Thus, our study identifies Stx2 as a novel regulator of phagocytosis and elucidates its mechanism for balancing phagocytic uptake and clearance.

## RESULTS

### Stx2 limits the ability of macrophages to engage and uptake IgG-opsonized particles

**Fig. 1.**
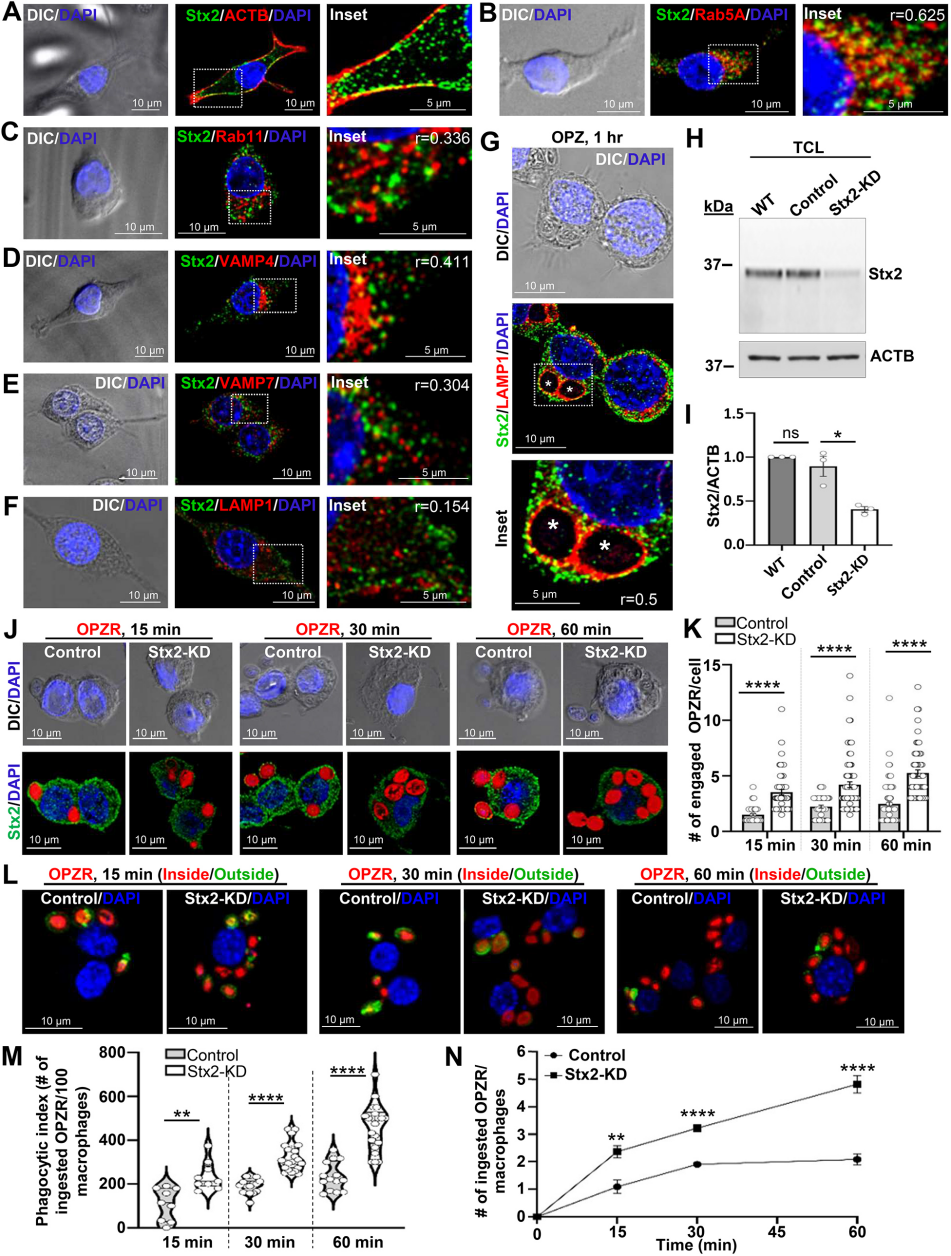
**Stx2 exhibits multiple subcellular distributions in macrophages, and inhibits engagement and uptake of IgG-opsonized particles.** (A–F) DIC and confocal images of endogenous Stx2 with endogenous (A) β-actin (ACTB), (B) RAB5A, (C) Rab11, (D) VAMP4, (E) VAMP7 and (F) LAMP1 at steady-state. (G) DIC and confocal images of RAW 264.7 macrophages fed with OPZ (white asterisks) for 1h and probed for endogenous Stx2 and LAMP1. Boxed areas enlarged in the insets. ‘*r*’ values show degree of colocalization. Scale bars: 10 μm (main images) and 5 μm (insets). DAPI denotes the nucleus. All images representative of three independent repeats. (H) Western blot of Stx2 and β-actin (ACTB, loading control) from RAW 264.7 macrophages (WT) and macrophages transduced with scrambled (sc) shRNA (control) or shRNAs targeted to Stx2 (Stx2-KD). (I) Quantified Stx2 band density normalized to ACTB (loading control). *N*=3 independent experiments. Results are mean±s.e.m. ns, not significant; *P<0.05. (J) DIC and stacked (three consecutive optical sections) confocal images from 15, 30 and 60 min OPZR-incubated macrophages also probed for Stx2. DAPI marks the nucleus. Scale bars: 10 μm. (K) Quantification of engaged OPZR per macrophage (15 min: control, *n*=61; Stx2-KD, *n*=82; 30 min: control, *n*=56; Stx2-KD, *n*=109; 60 min: control, *n*=111; Stx2-KD, *n*=86). *N*=3 independent experiments. Results are mean±s.e.m. ****P<0.0001. (L) Maximum intensity projection confocal images of inside-outside stained macrophages incubated with OPZR for 15, 30 and 60 min. Scale bars: 10 µm. (M,N) Quantification of (M) phagocytic index and (N) phagocytic rate for OPZR analyzed at 15 min (control, *n*=75; Stx2-KD, *n*=68), 30 min (control, *n*=273; Stx2-KD, *n*=344) and 60 min (control, *n*=165; Stx2-KD, *n*=190). *N*=3 independent experiments. M shown as violin plots with median and quartiles marked. For N, results are mean±s.e.m. **P<0.01, ****P<0.0001. All statistical tests were two-tailed unpaired Student's *t*-tests. See also Fig. S1.

To understand functions of Stx2 in macrophage phagocytosis, we first assessed the localization of endogenous Stx2 within the subcellular compartments of RAW 264.7 macrophages. Using confocal immunofluorescence imaging, at steady state, we detected predominant presence of Stx2 on plasma membrane (Fig. 1A) and on Rab5A-positive early endosomes (Fig. 1B; *r*=0.625). We also observed some moderate colocalization with Rab11-positive recycling endosomes (Fig. 1C; *r*=0.336) and VAMP4-positive compartments (Fig. 1D; *r*=0.411) that include trans-Golgi network, sorting and recycling endosomes (Shui et al., 2008; Tran et al., 2007). A moderate colocalization was also observed with VAMP7-positive late endosomes (Fig. 1E; *r*=0.304). Stx2 was poorly localized with the lysosome marker protein LAMP1 (Fig. 1F; *r*=0.154). To assess Stx2 localization on phagosomes, we imaged macrophages that had ingested IgG-opsonized zymosan particles (OPZ) for 1 h (Fig. 1G). Phagosomes fuse with lysosomes, and in the process acquire lysosomal membrane protein LAMP1. In accordance with an earlier report (Hackam et al., 1996), we observed significant colocalization (*r*=0.5) of Stx2 (green) with LAMP1 (red), which delineated phagosome membranes around the non-fluorescent OPZ (Fig. 1G). Collectively, these data demonstrate the varied intracellular distribution of Stx2 in macrophages.

To functionally characterize Stx2, we established Stx2-depleted (Stx2-KD) RAW 264.7 cell lines by stably expressing shRNAs targeted to mouse Stx2 (Fig. S1A,B). Among six clones (C1–C3, C5–C7) with varying knockdown (KD) efficiency (Fig. S1A,B), C1 showed the strongest depletion of Stx2 (∼70%; Fig. 1H,I) and was selected as the primary clone for all experiments. A parallel control cell line was generated by expressing nonspecific scrambled-shRNA (sc-shRNA) that did not affect expression of endogenous Stx2 (Fig. 1H,I). In order to examine impact of Stx2 depletion on phagocytosis, we initially incubated control and Stx2-KD macrophages with IgG-opsonized red-fluorescent latex beads of 2 µm diameter (OPFB-2) for 15 to 60 min (Fig. S1C). Differential interference contrast (DIC) and confocal imaging showed increased association of OPFB-2 to Stx2-KD macrophages at all the investigated time points (Fig. S1C,D). To assess whether this was a general phenomenon for IgG-opsonized particles, we exposed macrophages to opsonized zymosan red-fluorescent particles (OPZR) (Czibener et al., 2006). We also immunostained macrophages for Stx2 to directly correlate the change in particle association with Stx2 level (Fig. 1J). We observed a consistent surge in OPZR engagement in Stx2-depleted macrophages (15 min, ∼2.4-fold; 30 min, ∼1.9-fold; 60 min: ∼2.2-fold) (Fig. 1J,K), which is comparable to that seen with OPFB-2 (Fig. S1C,D). Stx2-KD thus seems to augment macrophage capacity for opsonized particle engagement.

To verify, whether the increased particle engagement translated into increased phagocytic uptake, we compared phagocytic index (number of ingested particles/100 macrophages) among control and Stx2-KD RAW 264.7 macrophages exposed to OPZR for 15 to 60 min (Fig. 1L,M). An inside–outside staining approach was adopted to discriminate internalized particles from external particles (Choy and Botelho, 2017). Quantification revealed a significant increase in phagocytic index in Stx2-depleted macrophages throughout the incubation period (15 min, ∼2.16-fold; 30 min, ∼1.69-fold; 60 min, ∼2.06-fold) (Fig. 1L,M). The rates of phagocytosis were also increased significantly (control: 2.4 OPZR/macrophage/60 min; Stx2-KD, 5.1 OPZR/macrophage/60 min) (Fig. 1N). To verify whether the observed phagocytic output is an outcome of clonal variation, we evaluated the OPZR phagocytic index in macrophages from independently generated C5 and C6 clones (Fig. S1E,F). Both clones showed increased phagocytosis, albeit to a lesser extent than C1 (Fig. 1M; Fig. S1E,F), and this effect correlated directly with Stx2 levels. Furthermore, control and wild-type macrophages showed equivalent phagocytosis competency (Fig. S1E,F). These results thus suggest that enhanced particle engagement and phagocytosis can be specifically attributed to the depletion of Stx2.

### Enhanced formation and expansion of phagocytic cups in Stx2-depleted macrophages

**Fig. 2.**
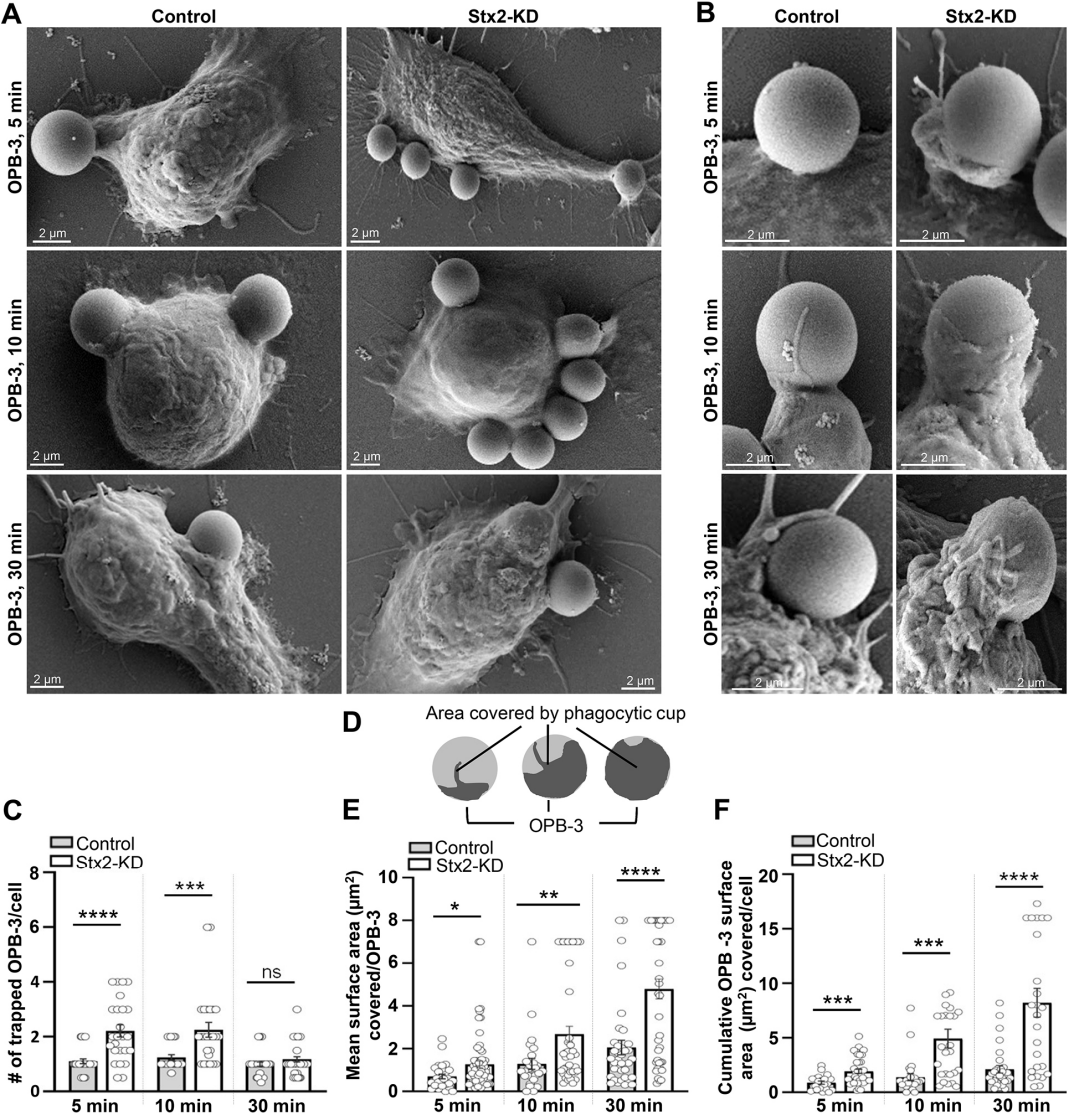
**Enhanced formation and expansion of phagocytic cups in Stx2-depleted macrophages.** (A) Representative scanning electron micrographs of RAW 264.7 macrophages incubated with OPB-3 for 5, 10 and 30 min. Scale bars: 2 μm. Additional set of images in Fig. S2. (B) Magnified micrographs show single phagocytic cups. Scale bars: 2 μm. All images representative of three independent repeats. (C) Quantification of the surface attached OPB-3 per macrophage (5 min: control, *n*=29; Stx2-KD, *n*=25; 10 min: control, *n*=23; Stx2-KD, *n*=25; 30 min: control, *n*=33; Stx2-KD, *n*=36). *N*=3 independent experiments. Results are mean±s.e.m. ns, not significant; ***P<0.001; ****P<0.0001. (D) Schematic diagram of partially ingested (shaded) OPB-3. Shaded areas were estimated to calculate the extent of phagocytic cup expansion. (E,F) Quantification of the (E) mean surface area (µm^2^) covered in individual OPB-3 (mean area of individual phagocytic cup) and (F) cumulative bead surface areas (µm^2^) wrapped with plasma membrane as sum of total plasma membrane expansion (5 min: control, *n*=17; Stx2-KD, *n*=22; 10 min: control, *n*=20; Stx2-KD, *n*=17; 30 min: control, *n*=19; Stx2-KD, *n*=23). *N*=3 independent experiments. Results are mean±s.e.m. ns, not significant; *P<0.05; **P<0.01; ***P<0.001; ****P<0.0001. All statistical tests were two-tailed unpaired Student's *t*-tests.

Engagement of IgG-opsonized particles with macrophages develops phagocytic cups for grasping phagocytic particles (Flannagan et al., 2012; Swanson, 2008). Expansion of phagocytic cups sequesters particles within vesicular phagosomes (Flannagan et al., 2012; Swanson, 2008). Stx2-KD enhances the engagement and uptake of IgG-opsonized particles (Fig. 1J–N). We therefore utilized scanning electron microscopy (SEM) to scrutinize alterations in macrophage surface morphology during phagocytosis of IgG-opsonized latex beads of 3.0 µm diameter (OPB-3). At an early time point (5 min) Stx2-KD macrophages exhibited an ∼2-fold increase in OPB-3 attachment (Fig. 2A,C), with clear emergence of phagocytic cups (Fig. 2A,B). The numbers of attached OPB-3 particles were increased by ∼1.8-fold after 10 min, accompanied by prominent appearance of phagocytic cups (Fig. 2A,B). By 30 min, there was a marked decrease in attached OPB-3 particles in both control and Stx2-KD macrophages, with the majority of the attached OPB-3 having been internalized (Fig. 2A,C).

For a quantitative assessment of phagocytic cup expansion, we measured the plasma membrane area (µm²) that surrounds the partially internalized OPB-3, as depicted by shaded regions in the schematic diagram (Fig. 2D). These values were expressed as the mean surface area per OPB-3 (Fig. 2E) and as the cumulative surface area (Fig. 2F) to compare both mean phagocytic cup sizes and total plasma membrane expansion between control and Stx2-KD macrophages. Analysis revealed an ∼1.8-fold increase in mean cup size after 5 min, an ∼2.67-fold increase after 10 min and an ∼2.33-fold increase after 30 min (Fig. 2E) in Stx2-KD macrophages. Similarly, cumulative membrane expansion was significantly enhanced in Stx2-KD macrophages, showing an ∼2.17-fold, ∼3.56-fold and ∼3.87-fold increase at 5, 10 and 30 min, respectively (Fig. 2F). Collectively, these data suggest that Stx2-depleted macrophages can expand plasma membrane uncontrollably for increased number of particle trapping and uptake.

### Stx2 curbs surface-expressed FcR by controlling receptor recycling

**Fig. 3.**
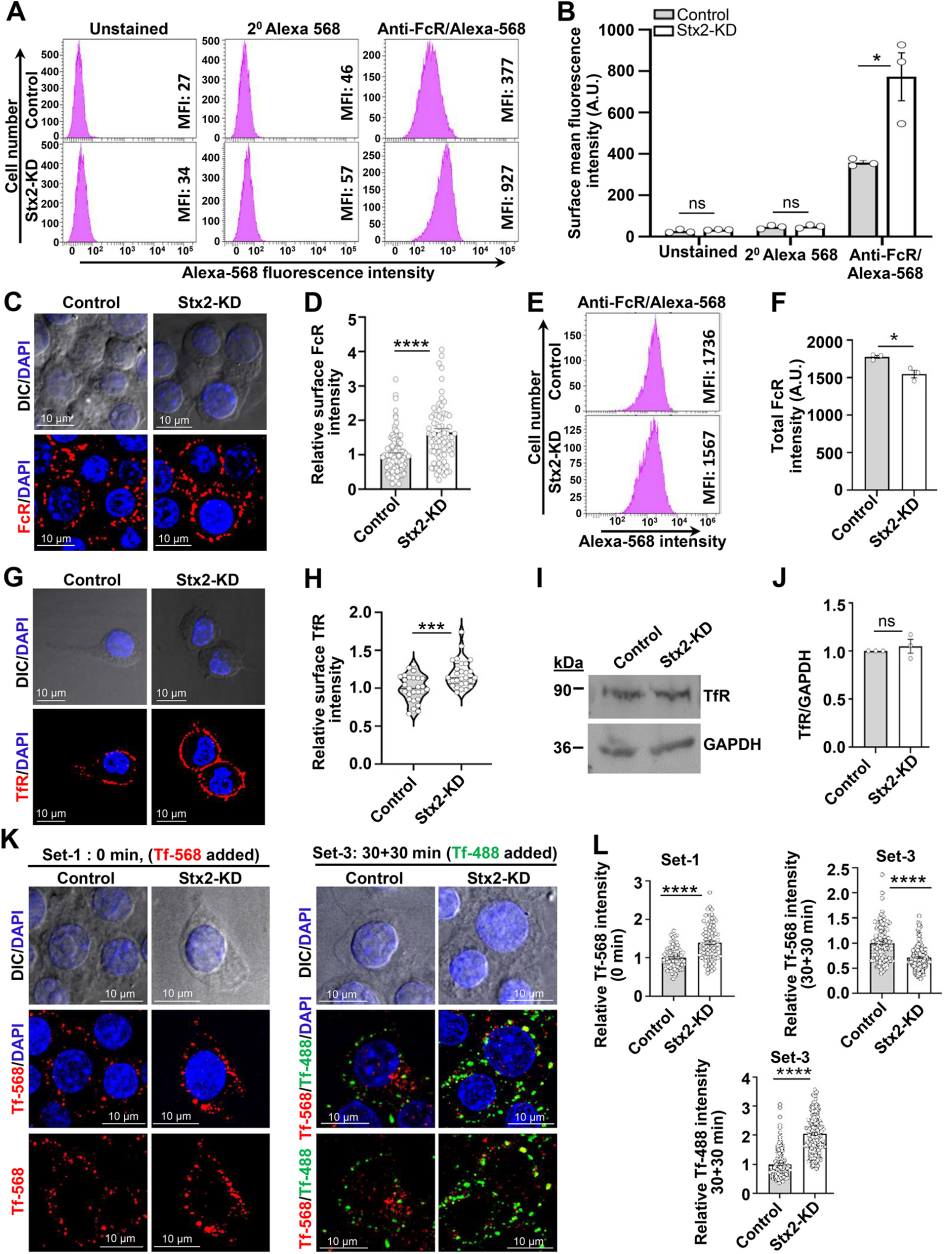
**Stx2 curbs surface-expressed FcR by controlling receptor recycling.** (A) FACS histograms with mean fluorescence intensity (MFI) of unlabeled, only secondary (2°) antibody-labeled and FcR-labeled macrophages. (B) Mean MFI values of three independent FACS histograms. Results are mean±s.e.m. ns, not significant; *P<0.05. FACS analysis of surface FcR for C5 and C6 macrophages are in Fig. S3A. (C) DIC and confocal images of surface FcR. DAPI marks the nucleus. Scale bars: 10 μm. (D) Quantified surface fluorescence intensity for FcR (control, *n*=105; Stx2-KD, *n*=78). *N*=3 independent experiments. Results are mean±s.e.m. ****P<0.0001. (E) FACS histograms for total FcR with MFI values. (F) Mean MFI values for total FcR from three independent FACS histograms. Results are mean±s.e.m. *P<0.05. (G) DIC and confocal images of surface TfR. Nuclei were labeled with DAPI. Scale bars: 10 μm. (H) Quantified surface TfR fluorescence intensity (control, *n*=27; Stx2-KD, *n*=27). *N*=3 independent experiments. Results shown as violin plots with median and quartiles marked. ***P<0.001. (I) Western blot of TfR and GAPDH (loading control) from macrophages. (J) Quantified TfR band density normalized to GAPDH. *N*=3 independent experiments. Results are mean±s.e.m. ns, not significant. (K) Tf-568-labeled surface TfR in resting macrophages (left, Set-1), and Tf-488 labeled surface recycled TfR (right, Set-3). (L) Quantification of surface Tf-568 fluorescence intensity (control, *n*=89; Stx2-KD, *n*=83) at rest from Set-1 (top left) and total (surface plus internal) Tf-568 intensity (top right) and surface TfR bound Tf-488 intensity (bottom) from Set-3, after recycling (control, *n*=100; Stx2-KD, *n*=155). *N*=3 independent experiments. Results are mean±s.e.m. ****P<0.0001. See also Fig. S3B,C for Tf-568 labeled TfR endocytosis (Set-2). All statistical tests were two-tailed unpaired Student's *t*-tests.

Phagocytic receptors engage phagocytic particles with phagocytes (Freeman and Grinstein, 2014). Stx2-depleted macrophages show heightened engagement of IgG-opsonized particles (Fig. 1J; Fig. 2A,C). We therefore assessed surface expression of IgG Fc region binding phagocytosis receptors FcR. Non-permeabilized macrophages were immunostained with FcR-specific antibodies to label surface receptors and subjected to FACS analysis that showed ∼2.3-fold increase in Stx2-KD macrophages (Fig. 3A,B). Analysis of macrophages from the C5 and C6 clones also showed a consistent rise in surface-expressed FcR (Fig. S3A). Consistently, non-permeabilized Stx2-KD macrophages produced an ∼1.7-fold higher fluorescence intensity for FcR in confocal imaging (Fig. 3C,D). To address whether this increased surface FcR abundance was an outcome of increased cellular expression or increased plasmalemmal delivery, we assessed total FcR content in permeabilized macrophages by FACS, which, by contrast, revealed a slight drop in Stx2-depleted macrophages (Fig. 3E,F). Consistent with the results for FcR, we observed higher surface expression of the iron transporter transferrin receptor (TfR) in Stx2-KD macrophages (Fig. 3G,H) despite no change in its total cellular expression (Fig. 3I,J). Surface delivery of FcR relies on recycling from its intracellular endosomal stores (Mellman et al., 1984). FcR and TfR take same recycling route to plasma membrane (Cox et al., 2000). Consequently, we evaluated TfR recycling to determine whether altered recycling contributed to increased surface receptor expression. TfR undergoes endocytosis upon binding to iron-bound transferrin (Tf), and after releasing iron, recycled to plasma membrane where Tf dissociates, enabling a new round of iron uptake (Baravalle et al., 2005). Using a slightly revised methodology (Sheff et al., 2002), we differentially labeled resting surface TfR with Alexa Fluor 568-conjugated transferrin (Tf-568; red fluorescence) and recycled TfR with Alexa Fluor 488-conjugated transferrin (Tf-488; green fluorescence) (Fig. 3K). Consistent with the TfR immunostaining results (Fig. 3G,H), resting Stx2-KD macrophages displayed increased surface binding of Tf-568 (Fig. 3K,L; Set-1). After 30 min of incubation, both control and Stx2-KD macrophages showed Tf-568 distribution throughout the cell with a concomitant drop in peripheral Tf-568 (Fig. S3B; Set-2), suggesting active endocytosis. A further 30 min incubation to allow sufficient TfR recycling and subsequent labeling of recycled TfR with Tf-488 showed a significant drop in red fluorescence intensity and a concurrent surge in green fluorescence intensity (binding of Tf-488) at the periphery (surface) of Stx2-KD macrophages (Fig. 3K,L; Set-3). Thus, these data suggest that elevated receptor recycling underlies increased surface expression of FcR for increased IgG-opsonized particle engagement.

### Stx2 prevents non-lytic compartment acquisition for phagocytic cup expansion and phagosome biogenesis

**Fig. 4.**
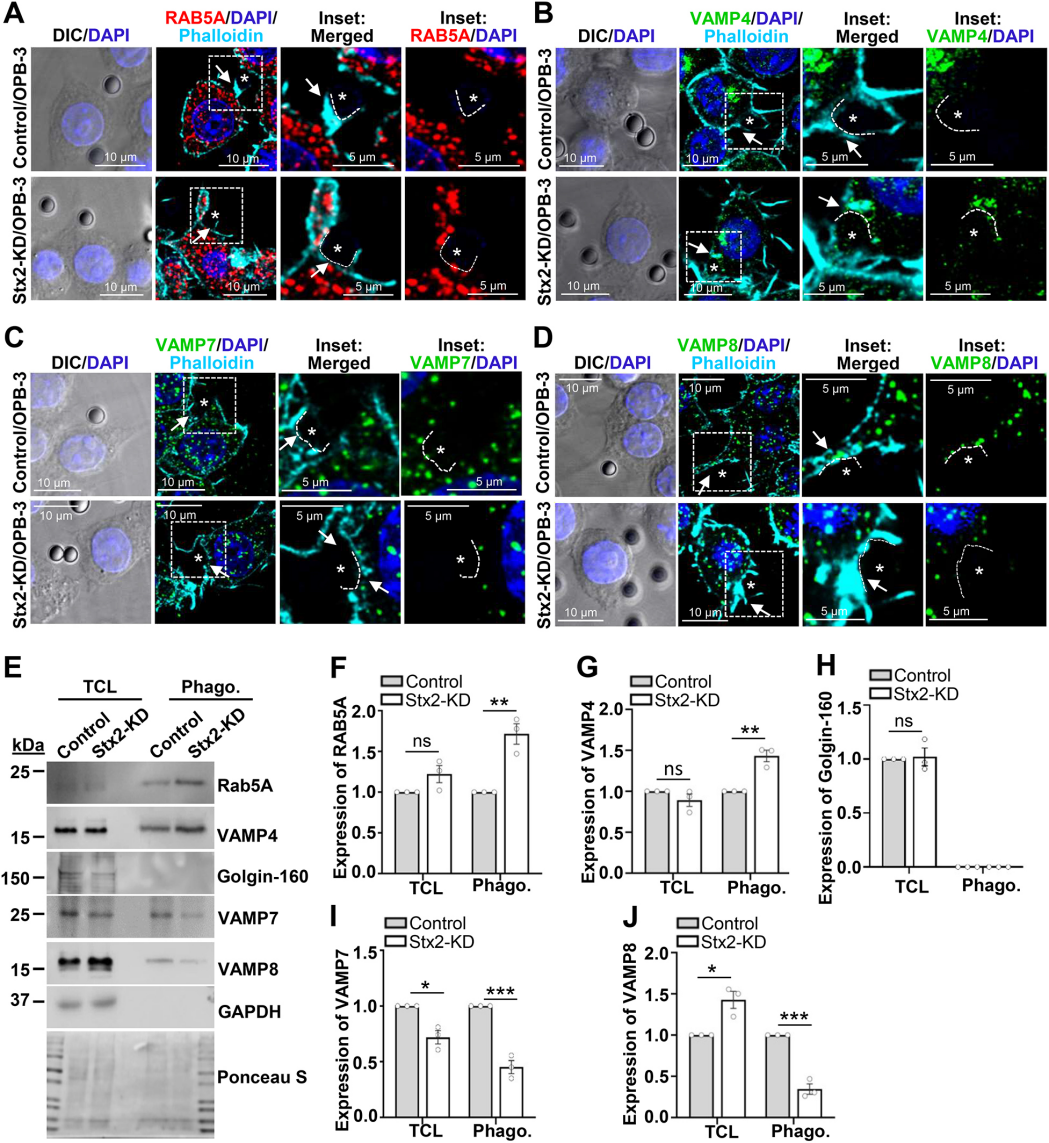
**Stx2 curtails acquisition of early endosomes and VAMP4-positive post-Golgi compartments for phagocytic cup expansion and phagosome biogenesis.** (A–D) DIC and confocal images of 15 min OPB-3 challenged macrophages probed for F-actin (phalloidin) and (A) Rab5A, (B) VAMP4, (C) VAMP7 and (D) VAMP8. DAPI shows the nuclei. White asterisks mark OPB-3-containing phagocytic cups with incomplete phalloidin lining (indicated by white arrows). Boxed regions enlarged in the insets, where positions of phagocytic cups are outlined by white dotted lines. Scale bars: 10 μm (main images) and 5 μm (inset). All images representative of three independent repeats. (E) Western blots of macrophage total cell lysates (TCL) and purified phagosomes (phago) from 30 min OPB-0.8 fed macrophages, probed for Rab5A, VAMP4, Golgin-160, VAMP7, VAMP8 and GAPDH (TCL loading control). Ponceau S-stained membrane shows uniform loading. (F–J) Quantified band density of (F) RAB5A, (G) VAMP4, (H) Golgin-160, (I) VAMP7 and (J) VAMP8 normalized to GAPDH (for TCL) or to Ponceau S-stained lanes (for phagosomes). *N*=3 independent experiments. Results are mean±s.e.m. ns, not significant; *P<0.05; **P<0.01; ***P<0.001. Images of nascent phagosomes are shown in Fig. S4. All statistical tests were two-tailed unpaired Student's *t*-tests.

Expansion of phagocytic cups for phagosome biogenesis requires adequate supply of membranes from intracellular reservoirs (Flannagan et al., 2012; Shui et al., 2008). We next aimed to identify and compare the contributions of specific intracellular compartments to phagosome biogenesis during early point of uptake. We imaged macrophages challenged with OPB-3 for 15 min to capture phagocytic cups, identified by incomplete phalloidin-stained F-actin rings (Krendel and Gauthier, 2022) surrounding OPB-3 particles, further indicated here by dotted white lines (Fig. 4A–D). We attempted to detect Rab5A as an early endosome marker (Wandinger-Ness and Zerial, 2014) and the SNARE protein VAMP4, which is a marker for recycling and sorting endosomes, and the trans-Golgi network (Cebrian et al., 2011). We also probed the late endosome-localized SNARE proteins VAMP7 (Braun et al., 2004) and lysosome-localized SNARE protein VAMP8 (Diao et al., 2015). Both Rab5A (Fig. 4A) and VAMP4 (Fig. 4B) showed increased delivery at the phagocytic cups of Stx2-KD macrophages (Fig. 4A,B). Interestingly, we observed reduced delivery of VAMP7 (Fig. 4C) and VAMP8 (Fig. 4D). We extended the study to examine these compartments in nascent phagosomes by imaging macrophages challenged with OPB-3 for 30 min (Fig. S4A–D). In line with the observations on phagocytic cups, nascent phagosomes (marked by F-actin outlining; Defacque et al., 2000) of Stx2-depleted macrophages exhibited an elevated presence for both Rab5A (Fig. S4A) and VAMP4 (Fig. S4B). The staining for VAMP7 and VAMP8 consistently remained depleted and appeared punctate and incomplete compared to that seen in control nascent phagosomes (Fig. S4C,D).

To further determine the amounts of these compartments in nascent phagosomes, we performed western blot analysis of purified phagosomes from macrophages exposed to OPB beads of 0.8 µm diameter (OPB-0.8) for 30 min. We observed an ∼1.7-fold increase in Rab5A, and ∼1.43-fold increase in VAMP4 content in Stx2-KD phagosomes (Fig. 4E–G). VAMP4 has been shown to traffic through recycling and sorting endosomes to enrich at the trans-Golgi network, and this membrane is known to be absent from FcR-induced phagosomes (Cebrian et al., 2011; Desjardins et al., 1994). Consistent with this, we could not detect the trans-Golgi-dwelling protein Golgin-160 (GOLGA3) (Fig. 4E,H) in phagosomes, suggesting that they have membrane contributions from VAMP4-positive post-Golgi compartments. The presence of VAMP7 and VAMP8 also remained persistently low in Stx2-KD phagosomes and were decreased by 55% and 65.5% respectively (Fig. 4E,I,J). Altogether, Stx2 curtails delivery of non-lytic early endosomes and VAMP4-positive post-Golgi compartments for phagocytic cup expansion and phagosome biogenesis.

### Increased lysosome content and diminished pro-cathepsin secretion in Stx2-depleted macrophages

**Fig. 5.**
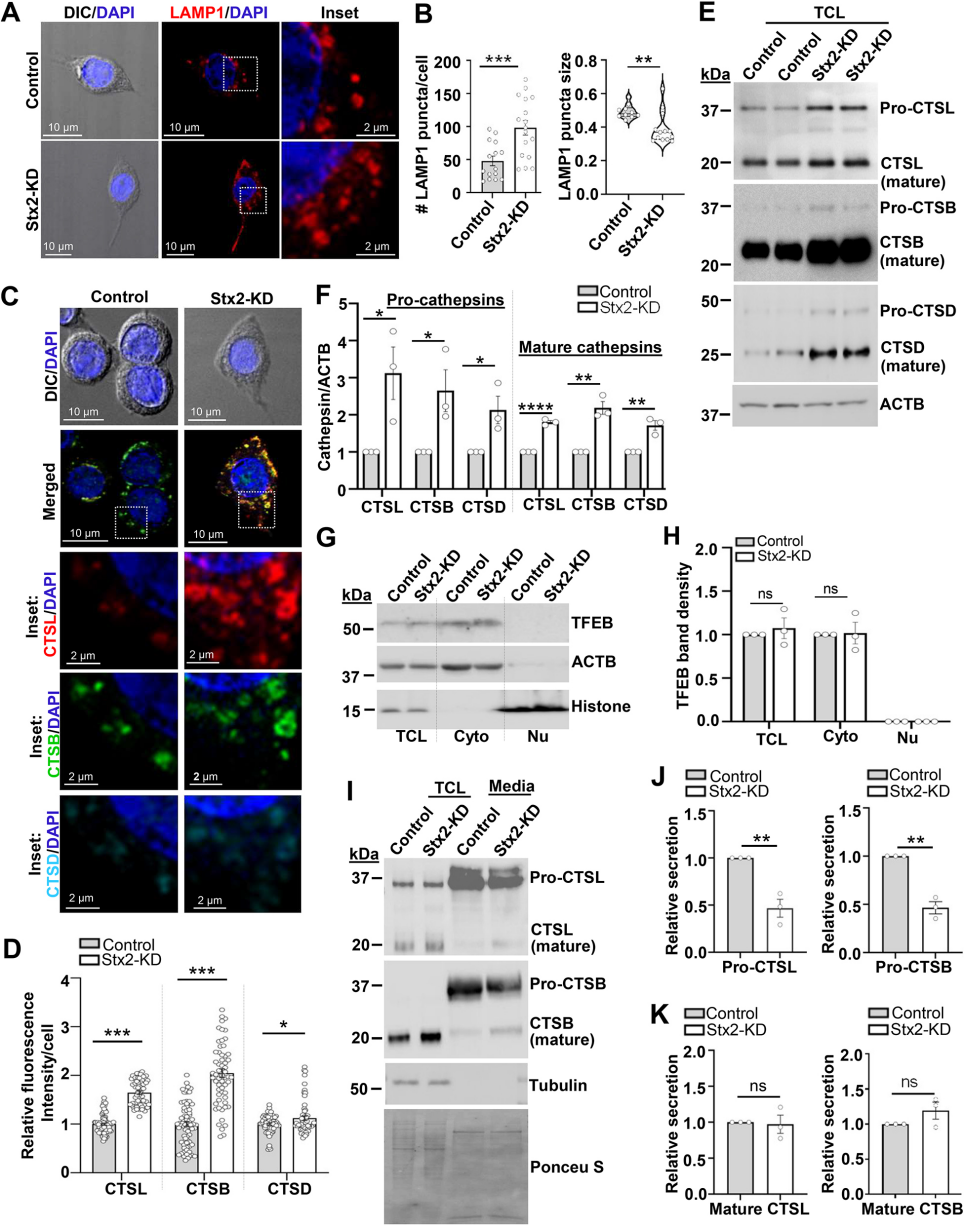
**Increased lysosome content couples with diminished pro-cathepsin secretion in Stx2-depleted macrophages.** (A) DIC and stacked (three consecutive optical sections) confocal images of LAMP1 immunostained macrophages. Boxed areas enlarged in inset. Nuclei labeled with DAPI. Scale bars: 10 µm (main images) and 2 µm (inset). (B) Macrophages quantified for number (left) and size (right) of LAMP1 puncta from control (*n*=54) and Stx2-KD (*n*=54) cells. *N*=3 independent experiments. Results are mean±s.e.m. (left) and violin plots with median and quartiles marked (right). **P<0.01; ***P<0.001. Analyses of macrophage LAMP1 puncta in C5 and C6 macrophages are in Fig. S5C–E. (C) DIC and confocal merged images for CTSL, CTSB and CTSD. Boxes are magnified and split into separate channels in inset. Scale bars: 10 µm (main images) and 2 µm (inset). (D) Quantified (control, *n*=73; Stx2-KD, *n*=56) fluorescence intensity for cathepsins. *N*=3 independent experiments. Results are mean±s.e.m. *P<0.05, ***P<0.001. (E) Western blots of macrophage total cell lysates (TCL) for CTSL, CTSB, CTSD and ACTB (loading control). (F) Quantified band density for pro- and mature forms of CTSL, CTSB and CTSD, normalized to ACTB. *N*=3 independent experiments. Results are mean±s.e.m. *P<0.05, **P<0.01, ****P<0.0001. (G) Western blot detection for TFEB in nuclear (Nu) and cytosolic (Cyto) fractions. ACTB was used as a marker as well as a loading control for TCL and Cyto fraction. Histone served as a marker and loading control for Nu fractions. (H) TFEB band density normalized to ACTB for TCL and Cyto fractions and to histone for Nu fractions. *N*=3 independent experiments. Results are mean±s.e.m. ns, not significant. (I) Western blot detection of macrophage secreted pro- and mature CTSL and CTSB. Tubulin and Ponceau S-stained membranes were used as loading control for TCL and media, respectively. (J,K) Quantified secreted (J) pro-CTSL (left) and pro-CTSB (right) and (K) mature CTSL (left) and mature CTSB (right). TCL bands were normalized to tubulin and media bands were normalized to Ponceau S-stained lanes. *N*=3 independent experiments. Results are mean±s.e.m. ns, not significant. **P<0.01. All statistical tests were two-tailed unpaired Student's *t*-tests. See also Fig. S5.

Intrigued by the heightened expression of VAMP8 (Fig. 4E,J), a lysosomal SNARE protein (Diao et al., 2015), we postulated that depletion of Stx2 might also augment lysosome content in macrophages. To investigate, we immunostained macrophages for the lysosome membrane protein LAMP1 for direct visualization of lysosomes, which rose in number by ∼1.7-fold (Fig. 5A,B). Furthermore, western blot analysis validated an ∼2-fold increased abundance of LAMP1 in Stx2-depleted macrophages (Fig. S5A,B). Interestingly, the sizes of the puncta were relatively smaller compared to those in control macrophages (Fig. 5B). Verification on independently generated C5 and C6 clones also showed a consistent increase in number but reduction in sizes of LAMP1 puncta (Fig. S5C–E). Expression of major lysosomal hydrolases (Turk et al., 2000), such as cathepsin L (CTSL), cathepsin B (CTSB) and cathepsin D (CTSD) were also higher in Stx2-depleted macrophages (Fig. 5C,D). Western blots confirmed the increase in both premature (pro-cathepsins) and catalytically active mature cathepsins (Fig. 5E,F). Given that cathepsins are active in acidic (low pH) conditions, we assessed lysosome acidification using the acidotropic dye LysoTracker Red DND-99, which preferentially accumulates in low-pH vesicles, such as lysosomes (Freundt et al., 2007). FACS analysis revealed an ∼1.6-fold increase in the mean fluorescence intensity (MFI) in Stx2-depleted macrophages (Fig. S5F). Additionally, confocal imaging revealed a notable increase in abundance as well as in intensity (∼1.8-fold higher MFI) of red fluorescent puncta upon Stx2 depletion (Fig. S5G,H). These findings indicate effective and increased lysosome acidification in Stx2-KD macrophages. To determine the fusion and degradation competency of lysosomes, we next assessed basal macroautophagy (hereafter autophagy). Autophagy captures intracellular constituents in phagosome vesicles (marked by LC3-II, the lipidated version of LC3-I), that subsequently fuse with lysosomes for content degradation (Mizushima et al., 2011). An elevated basal autophagic flux, as evidenced by heightened LC3-II to LC3-I ratio and concurrent degradation of autophagy substrate protein p62 (also known as SQSTM1) (Fig. S5I–K) further attests increase in functional and fusion competent lysosomes. (Klionsky et al., 2016). In order to explain increased lysosome contents, we examined the status of transcription factor EB (TFEB), the master regulator for lysosome biogenesis (Sardiello et al., 2009; Settembre et al., 2011). TFEB expression was similar in both control and Stx2-KD macrophages (Fig. 5G,H). Moreover, TFEB was undetectable in nuclear fraction and remained confined to the cytosol (Fig. 5G,H), suggesting that it has no involvement in the increased number of lysosomes in Stx2-KD macrophages. Interestingly, secretion assays for cathepsins revealed a specific reduction in pro-cathepsin levels in the culture medium (Fig. 5I–K). The lack of detectable cytosolic tubulin in the medium indicates absence of membrane leakage or nonspecific release by cell demise. Moreover, the comparable levels of mature cathepsins confirmed selective impairment in procathepsin secretion (Fig. 5K). We hypothesize that increased retention of pro-cathepsins can lead to increased transition to mature cathepsin containing lysosomes to accumulate within Stx2-depleted macrophages.

### Stx2 is required for phagosome maturation through acquisition of late endosomes or lysosomes and phagosome acidification

**Fig. 6.**
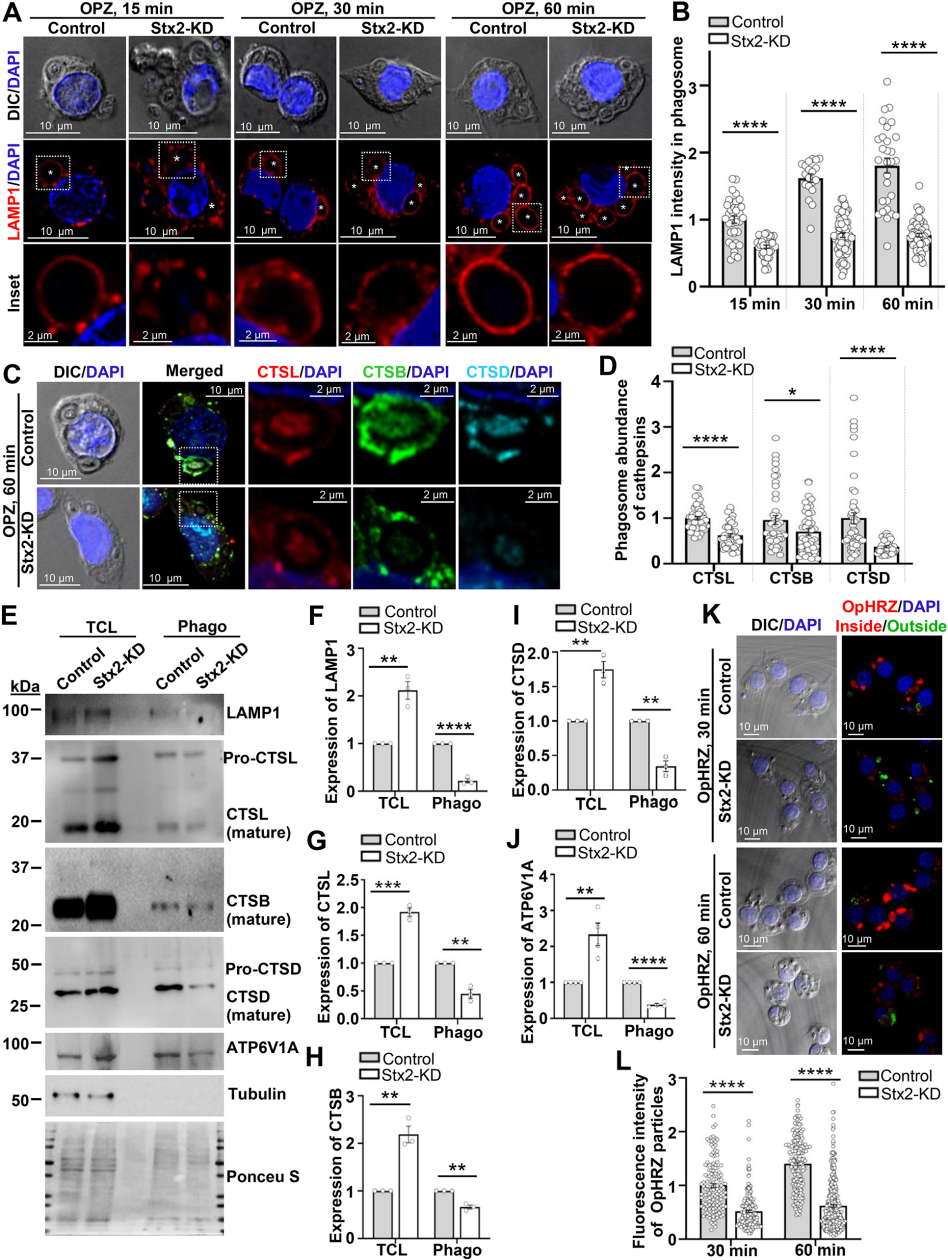
**Stx2 is required for macrophage phagosome maturation and acidification.** (A) DIC and confocal images of OPZ-fed macrophages immunostained for LAMP1. White asterisks show the OPZ-containing phagosomes. Boxed regions magnified in the inset. DAPI marks the nuclei. Scale bars: 10 µm (main images) and 2 μm (inset). (B) Quantification (15 min: control, *n*=35; Stx2-KD, *n*=42; 30 min: control, *n*=29; Stx2-KD, *n*=68; 60 min: control, *n*=29; Stx2-KD, *n*=53) of the phagosomal LAMP1 fluorescence intensity. *N*=3 independent experiments. Results are mean±s.e.m. ****P<0.0001. (C) DIC and confocal images of 60 min OPZ-fed macrophages immunostained for CTSL, CTSB and CTSD. Boxed areas are magnified and shown as separate channels in inset. DAPI labeled the nuclei. Scale bars: 10 µm (main images) and 2 μm (inset). (D) Quantification (control, *n*=46; Stx2-KD, *n*=43) of the phagosomal fluorescence intensity for CTSL, CTSB and CTSD. *N*=3 independent experiments. Results are mean±s.e.m. *P<0.05, ****P<0.0001. (E) Western blots of macrophage total cell lysates (TCL) and purified phagosomes (phago) probed for LAMP1, CTSL, CTSB, CTSD, ATP6V1A and tubulin (loading control for TCL and negative control for ‘phago’). Ponceau S-stained membrane shows uniform loading. (F–J) Quantified band density of (F) LAMP1, (G) CTSL, (H) CTSB, (I) CTSD and (J) ATP6V1A, normalized to tubulin (for TCL) or Ponceau S-stained lanes for phagosomes. *N*≥3 independent experiments. Results are mean±s.e.m. **P<0.01; ***P<0.001; ****P<0.0001. (K) DIC and confocal images of 30 and 60 min OpHRZ-fed macrophages. DAPI used to visualize nuclei. Scale bars: 10 µm. (L) Quantification of fluorescence intensity (higher intensity indicates lower pH) of internalized (red) OpHRZ particle (30 min: control, *n*=113; Stx2-KD, *n*=66; 60 min: control, *n*=129; Stx2-KD, *n*=131). *N*=3 independent experiments. Results are mean±s.e.m. ****P<0.0001. All statistical tests were two-tailed unpaired Student's *t*-tests.

The extensive abundance of phagosomes (Fig. 1L–N) and lysosomes (Fig. 5) but reduced delivery of late endosomes and lysosomes to phagocytic cups (Fig. 4C,D) and nascent phagosomes (Fig. S4C,D; Fig. 4E,I,J) in Stx2-depleted macrophages prompted us to investigate possible defect in phagosome maturation. To delineate, we exposed macrophages to OPZ for 15 to 60 min (Fig. 6A). In line with our prior observations (Fig. 1L–N), Stx2-KD macrophages engulfed more OPZ particles throughout the experimental timeframe (Fig. 6A). We observed a gradual and prominent increase of LAMP1 fluorescence intensity around the control phagosomes, compared to phagosomes of Stx2-depleted macrophages (Fig. 6A,B). Concurrently, the presence of CTSL, CTSB and CTSD also remained low in Stx2-depleted phagosomes (Fig. 6C,D). These observations were validated using purified phagosomes from macrophages incubated with OPB-0.8 for a longer period (1 h uptake and 30 min maturation). We obtained a reduced amount of LAMP1 as well as catalytically active mature CTSL, CTSB and CTSD in Stx2-depleted phagosomes (Fig. 6E,F). Consistent with the disrupted secretion of pro-cathepsins (Fig. 5I,J), we also observed a reduced presence of pro-cathepsins in Stx2-KD phagosomes, suggesting that Stx2 is also required for the phagosomal incorporation of pro-cathepsin-bearing vesicles (Yadati et al., 2020). Given that acidic pH is required for maturation and activity of cathepsins (Laurent-Matha et al., 2006; Turk et al., 1999) for degradation of ingested particles (Di et al., 2006), we additionally probed for the phagosome-acidifying vacuolar ATPase (Hackam et al., 1997) subunit ATP6V1A. The decreased presence of ATP6V1A in Stx2-depleted phagosomes (Fig. 6E,J) suggests impaired phagosomal acidification. To confirm this, we allowed macrophages to engulf opsonized pHrodo red zymosan particles (OpHRZ), whose fluorescence increases with reduction in pH (increasing acidity) (Fig. 6K). Internalization of OpHRZ was confirmed by inside (red) and outside (green) staining. Internalized OpHRZ in control cells produced bright red fluorescence upon 30 min that further increased at 60 min (Fig. 6K,L). However, the fluorescence intensity of OpHRZ remained consistently dim within Stx2-KD macrophages (Fig. 6K,L). The MFI of the OpHRZ particles also remained significantly lower than that for controls at both 30 min and 60 min (Fig. 6L). Based on these findings, we conclude that Stx2 is an essential for maturation and acidification of phagosomes in macrophages.

### Stx2-depleted macrophages exhibit increased accumulation of intact bacteria through unabated uptake and impaired clearance

**Fig. 7.**
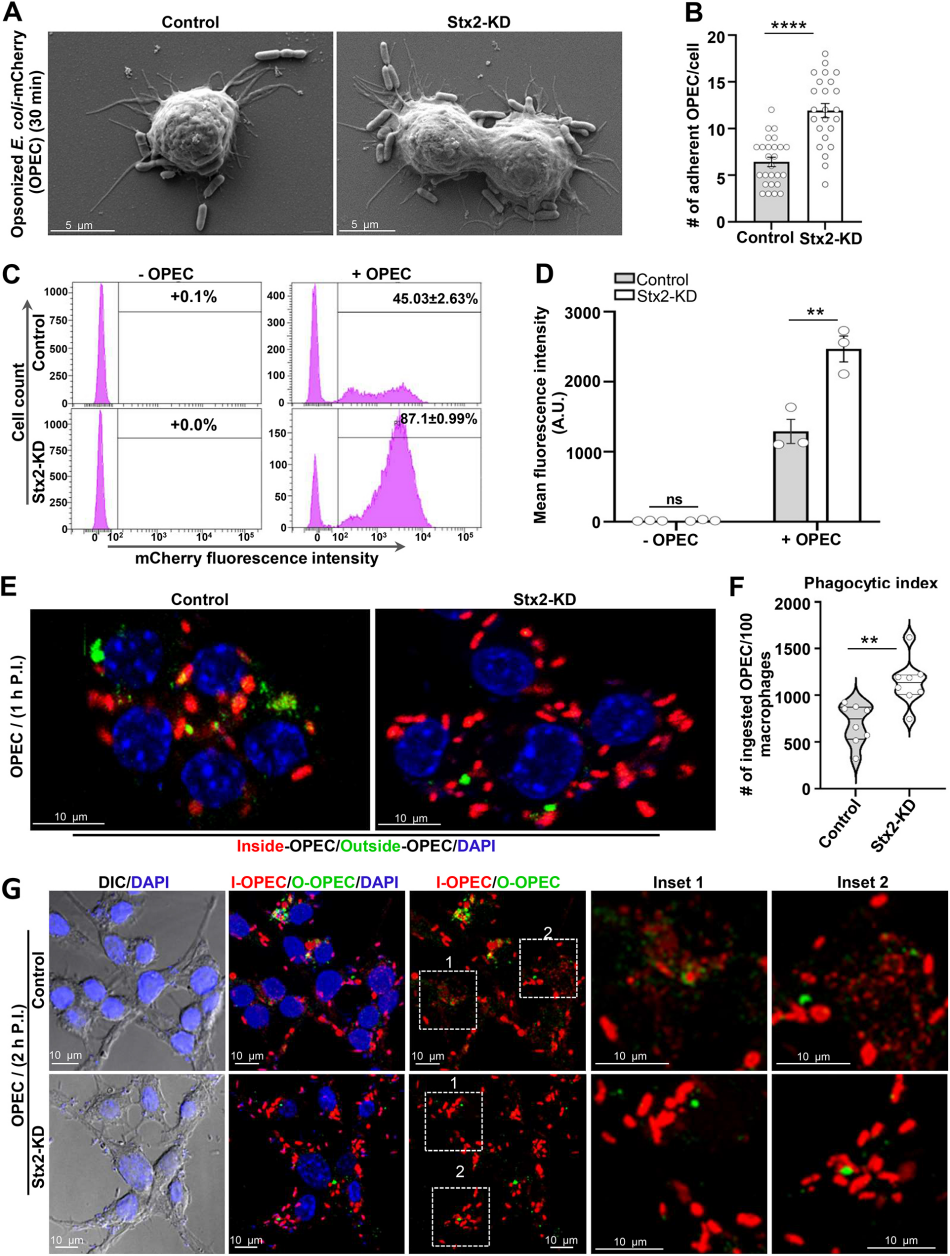
**Uncontrolled uptake of IgG-opsonized *E. coli* coupled with impaired clearance leads to increased accumulation of intact bacteria in Stx2-depleted macrophages.** (A) Representative SEM images of macrophages challenged with IgG-opsonized mCherry-expressing *E. coli* (OPEC) for 30 min. (B) Quantification of the number of surface adherent OPEC per macrophage (control, *n*=26; Stx2-KD, *n*=24). *N*=3 independent experiments. Results are mean±s.e.m. ****P<0.0001. (C) Representative FACS histograms showing extent of OPEC engagement to macrophages shown as shift in fluorescence intensity. (D) Mean MFI (OPEC/cell) from three independent FACS Results are mean±s.e.m. ns, not significant; **P<0.01. (E) Representative confocal maximum intensity projection images of macrophages inside (red)-outside (green) stained for OPEC. DAPI was used to stain the nuclei. Scale bars: 10 µm. (F) Quantification (control, *n*=141; Stx2-KD, *n*=141) of phagocytic index for OPEC. *N*=3 independent experiments. Results shown as violin plots with median and quartiles marked. **P<0.01. (G) Representative DIC and confocal maximum intensity projection images of 2 h (1 h uptake+1 h maturation) OPEC-incubated macrophages subjected to inside-outside staining for OPEC. Nuclei were stained with DAPI. Boxed areas are magnified in the inset. Scale bars: 10 μm. Phagocytic uptake and degradation by C5 and C6 macrophages for OPEC are in Fig. S7A–C. All statistical tests were two-tailed unpaired Student's *t*-tests.

The antibacterial efficacy of macrophages relies on their ability to engulf bacteria into phagosomes and subsequently their clearance by enzymatic degradation within phagolysosomes (Fairn and Grinstein, 2012). Increased phagocytic uptake but delayed phagosome maturation on Stx2 depletion (Figs 1 and 6) led us to assess the Stx2 effect on bacterial phagocytosis and degradation. We used IgG-opsonized mCherry-expressing *Escherichia coli* (OPEC). Stx2-depleted macrophages trapped high numbers (∼1.86-fold) of OPEC over control RAW cells (Fig. 7A,B). FACS analysis unveiled that 87.1±0.99% (mean±s.e.m) of Stx2-KD macrophages were associated with OPEC compared to only 45.03±2.63% in control macrophages (Fig. 7C). The MFI, serving as an indicator of the degree of association of fluorescent particles per individual cell, was ∼1.91-fold higher in Stx2-KD cells (Fig. 7D). Fluorescence imaging of OPEC-incubated macrophages subjected to inside–outside staining revealed an ∼1.63-fold rise in the phagocytic index in Stx2-KD macrophages (Fig. 7E,F). Longer incubation of OPEC (1 h internalization plus 1 h phagosome maturation) to examine macrophage digestion and clearance efficacy showed that the majority of the internalized OPEC became fragmented in control macrophages (Fig. 7G) whereas they remained undigested in Stx2-depleted macrophages, leading to an increased build-up of intact bacteria (Fig. 7G). Assessment of phagocytic index for OPEC in C5 and C6 macrophages confirmed a consistent increase (Fig. S7A,B), as observed in C1 macrophages (Fig. 7E,F). Similar to C1, macrophages from C5 and C6 also displayed impaired OPEC degradation and increased accumulation of undigested bacteria (Fig. S7C). These results demonstrate a reproducible phenotype across independently generated Stx2-KD clones. Stx2 is thus crucial for controlling bacterial clearance by phagocytosis in macrophages.

## DISCUSSION

**Fig. 8.**
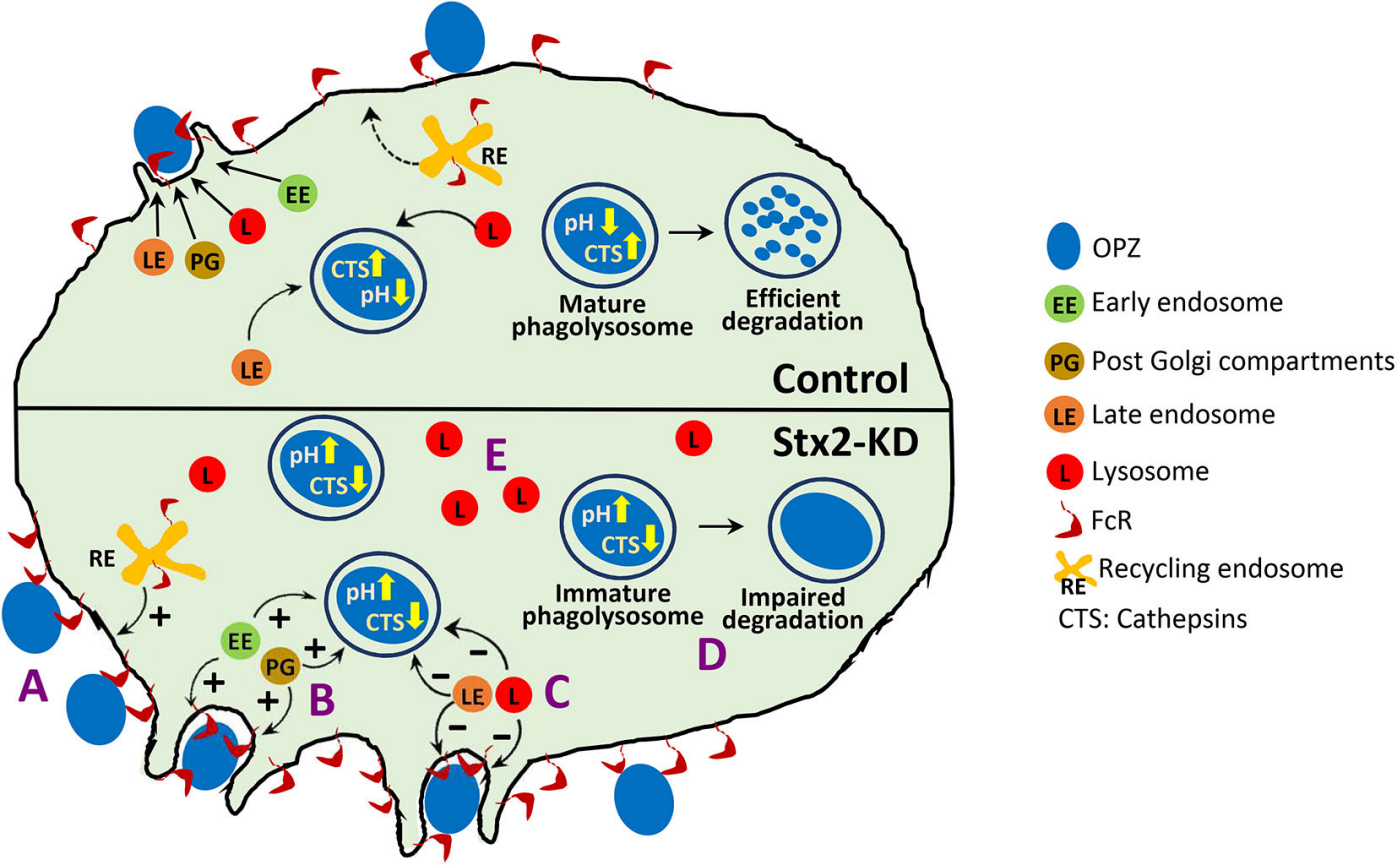
**A simplified model of how Stx2 depletion alters macrophage phagocytosis through precise control over vesicle trafficking.** Stx2-KD (A) elevates receptor recycling to augment surface FcR expression and engagement of IgG opsonized particles (OPZ), (B) increases acquisition of early endosomes (EE) and VAMP4-positive post-Golgi compartments (PG) by phagocytic cups and nascent phagosomes, (C) decreases acquirement of late endosomes (LE) and lysosomes (L) by phagocytic cups and nascent phagosomes, (D) impairs phagosome maturation and degradation ability by reducing attainment of cathepsin hydrolases (CTS) and acidity (higher pH) and (E) raises abundance of lysosomes.

In this study, we characterized the role of the SNARE protein Stx2 in macrophage phagocytosis for the very first time. We found that Stx2 coordinates the trafficking of multiple intracellular compartments in both basal states as well as during phagocytosis. Collectively, Stx2-regulated trafficking contributes to balancing phagocytic uptake and clearance of IgG-opsonized particles in macrophages (Fig. 8). This is corroborated by the wide distribution of Stx2 on the plasma membrane, early and late endosomes, VAMP4-positive compartments and phagosomes, which are directly involved in phagocytosis (Flannagan et al., 2012; Shui et al., 2008). On depletion of Stx2 (Stx2-KD), we noticed aberrant engagement and uptake of IgG-opsonized particles (Fig. 8). This enhanced engagement is attributed to higher surface expression of FcR in resting macrophages, which is an outcome of general increase in receptor recycling, whereas the total levels of receptors (FcR and TfR) remained similar upon Stx2-KD. Increased FcR engagement explains the increased formation of phagocytic cups, which requires the FcR–actin axis for plasma membrane remodeling (Swanson and Hoppe, 2004).

Expansion of phagocytic cups in forming phagosomes and maturation of phagosomes into phagolysosomes requires regulated acquisition of early endosomes, recycling endosomes, late endosomes and lysosomes mediated by SNARE proteins (Bajno et al., 2000; Braun et al., 2004; Czibener et al., 2006; Flannagan et al., 2012; Hackam et al., 1998). Stx2-depleted macrophages exhibited dysregulated expansion of phagocytic cups that was accompanied by enhanced delivery of early endosomes and VAMP4-positive compartments, leading to increased biogenesis of phagosomes. Previous proteomic and western blot analysis have confirmed abundant presence of VAMP4 in phagosomes (Shui et al., 2008), although its primary residence is in the trans-Golgi networks, and membrane from this does not take part in IgG-opsonized particle phagocytosis (Cebrian et al., 2011; Desjardins et al., 1994; Steegmaier et al., 1999). Our study also could not detect trans-Golgi marker protein in phagosomes. Given that VAMP4 traffics to trans-Golgi through sorting and recycling endosomes (Tran et al., 2007), phagosomal delivery might be enhanced for these compartments. As we could not confirm this with specific markers, we kept this as ‘VAMP4-positive post Golgi’ compartments.

Another intriguing finding from our study is that Stx2 depletion also increases macrophage lysosome contents. TFEB is the most prominent element that increases lysosomal protein synthesis for lysosome biogenesis (Settembre et al., 2011). We could not detect TFEB in the nucleus in Stx2-KD macrophages, suggesting TFEB-independent mechanisms. After synthesis in the ER, pro-cathepsins from trans-Golgi travel through various endosomal compartments that convert into late endosomes and fuse with lysosomes for full maturation of cathepsins (Yang and Wang, 2021). Considering that Stx2 is required for phagosomal hydrolase acquisition, we argue that Stx2 might also be required for hydrolase secretion. We observed a specific reduction in the secretion of pro-cathepsins in Stx2-KD macrophages, whereas the secretion of mature forms remained comparable. Impaired exocytosis is known to increase intracellular vesicle accumulation in both macrophages (Niedergang et al., 2003) and in non-immune cells (Dolai et al., 2016). We believe that Stx2 depletion increased pro-cathepsin-carrying vesicle flux for increased production of lysosomes (Yang and Wang, 2021). Interestingly, the lysosomes in Stx2-KD macrophages are smaller in size. V-ATPase is known to mediate lysosomal fission (Baars et al., 2007) and its increased expression in Stx2-KD macrophages could increase lysosomal fission. Although this could partly explain increased lysosome content (with more numbers and smaller in size) in Stx2-KD macrophages, the specific mechanisms that drive elevated expression of LAMP1 and VAMP8, and downregulation of VAMP7 might be distinct, and require further investigation for complete clarification. Furthermore, efficient acidification, as indicated by increased accumulation of the acidotropic dye lysotracker red (Freundt et al., 2007), coupled with elevated basal autophagy flux, which requires autophagosome–lysosome fusion and subsequent degradation, demonstrates that the lysosomes in Stx2-KD macrophages are catalytically active and fusion competent. However, this observation also indicates an increased cellular catabolic state (Rabinowitz and White, 2010) in Stx2-KD macrophages. Elevated autophagy generates ATP, which has been shown to promote receptor recycling (Podbilewicz and Mellman, 1990) and might be an additional factor contributing to enhanced receptor recycling in Stx2-KD macrophages. Thus, Stx2 is also crucial in regulating macrophage lysosome content for catabolic homeostasis (Settembre and Perera, 2024). Intriguingly, despite having abundant fusion competent and catalytically active lysosomes, Stx2-KD impaired phagosome acquisition of lysosomes and late endosomes and of the phagosome-acidifying V-ATPase. Collectively, Stx2-KD produced phagosomes that are less acidic (higher pH) and contained reduced levels of LAMP1 and cathepsins, which hence impaired biogenesis of phagolysosomes. The higher pH is known to diminish processing of pro-cathepsins and activity of mature cathepsins (Di et al., 2006; Laurent-Matha et al., 2006), which here resulted in impaired phagolysosomal degradation and clearance efficacy for ingested *E. coli* and increased retention of undamaged bacteria in Stx2-KD macrophages.

But how can Stx2 depletion selectively upregulate acquisition of some compartments but inhibit others? Previous works on Stx2 have disclosed its dual role in membrane fusions. For instance, in mucin-secreting goblet cells (Wojnacki et al., 2023) and in rat kidney cells (Low et al., 2003), Stx2 pairs up with VAMP8 to mediate membrane fusions. Conversely, in zymogen-secreting pancreatic acinar cells (Dolai et al., 2018) and in insulin-secreting pancreatic β-cells (Kang et al., 2022; Zhu et al., 2017), Stx2 acts as an inhibitory (i)-SNARE by binding with VAMP8 and VAMP2, and thereby depriving other cis-syntaxins in forming fusogenic SNAREpins. The increased delivery of early endosomes (Rab5A) and VAMP4-positive post-Golgi compartments to phagocytic cups of Stx2-KD macrophages and their increased presence on nascent phagosomes suggests that Stx2 acts as an i-SNARE during the acquisition of non-lytic compartments for phagosome biogenesis. The i-SNARE activity of Stx2 also extends to the fusion of receptor-bearing vesicles with plasma membrane as indicated by increased surface expression of receptors in Stx2-depleted macrophages. However, Stx2 appears to act as a fusogenic SNARE for the fusion of lytic compartments as suggested by the reduced acquisition of late endosomes and lysosomes during biogenesis and maturation of phagosomes, and by the decreased exocytosis of pro-cathepsin-containing vesicles. However, the identification of partner SNAREs and the mechanisms that regulate the contradictory behavior of Stx2 need further investigation. Alternatively, the increased Rab5 and VAMP4 levels in Stx2-KD phagosomes might result from prolonged retention due to defective Rab conversion or impaired NSF ATPase (that disassemble post-fusion SNAREpins for SNARE recycling) activity upon Stx2 depletion (Haas, 2007; Jahn and Scheller, 2006). Further studies are also needed to test these possibilities.

Nonetheless, our work unequivocally demonstrates the crucial involvement of Stx2 in macrophage phagocytosis. The opposing role of Stx2 in preventing excess uptake and concurrent promotion in lysosome-directed clearance of engulfed particles is unique among all known macrophage SNAREs. This balancing act of Stx2 could have far reaching physiological importance. Stx2 could be targeted to modulate phagocytosis plasticity for controlling hyperactive and diminished phagocytosis-related pathogenesis (Al-Samkari and Berliner, 2018; Butler et al., 2021; Kleinberger et al., 2014; Muñoz et al., 2010). It would be interesting to see the status and functions of macrophage Stx2 in such pathogenesis.

## MATERIALS AND METHODS

### Antibodies and reagents

Antibodies used for this study are enlisted in Table S1. Reagents used are mentioned in Table S2. Plasmids and oligonucleotides used are stated in Table S3. Any other materials, unless specified otherwise, were from Sigma-Aldrich.

### Macrophage cell culture

Authentic murine RAW 264.7 macrophage cells (ATCC, catalog #TIB-71) were grown in a humidified CO_2_ (5%) incubator at 37°C in complete Dulbecco's modified Eagle's medium (DMEM; Thermo Fisher Scientific, catalog #12100061), i.e. DMEM supplemented with 10% heat-inactivated fetal bovine serum (FBS; Avantor, catalog #97068-085), 100 U/ml of penicillin-streptomycin and 2 mM L-glutamine (complete DMEM).

### Generation of stable Stx2-knockdown and scrambled shRNA-expressing macrophages

Overnight cultures of passage 5 RAW 264.7 macrophage cells at ∼50% confluency were mixed with 5 µg/ml polybrene (Sigma-Aldrich, catalog #TR-1003) 1 h prior to infection with lentiviral particles [multiplicity of infection (MOI): 5] expressing either non-specific scrambled (sc) shRNA (Santa Cruz Biotechnology, catalog #sc-108080) to obtain control cells or expressing mouse Stx2-shRNAs (Santa Cruz Biotechnology, catalog #sc-41327-V) to obtain syntaxin-2-knockdown cells (Stx2-KD), following the manufacturer's guidelines. Virus and polybrene-containing media were replaced with fresh complete DMEM at 12 h after infection, and cells were allowed to grow for another 24 h before seeding onto 100 mm dishes. Puromycin (Sigma-Aldrich, catalog #P7255) was added to the culture medium at 5 µg/ml to select the transduced colonies. Six puromycin resistant colonies (C1, C2, C3, C5, C6 and C7) from three independent transduction (C1, C2 and C3 from the first transduction; C5 from the second transduction; and C6 and C7 from the third transduction) were retrieved individually using cloning cylinders (Sigma-Aldrich, catalog #Tr-1004) and maintained under selection pressure. The expression level of Stx2 was verified by western blotting, and the chosen control and Stx2-KD clones were propagated, frozen in liquid nitrogen, and considered as a new ‘Passage 1’. C1 was used to conduct all the experiments for this study, whereas C5 and C6 were used to measure clonal variations of fundamental observations (phagocytosis, surface FcR expression, lysosome content, bacterial uptake and degradation). All experiments were conducted using cells below 10 passages to minimize potential alterations due to prolonged culturing.

### Generation of mCherry-expressing *E. coli*

The mCherry sequence was amplified from the mCherry–TFR-20 plasmid (Addgene #55144) using forward primer: 5′-GCGCGGATCCATGGTGAGCAAGGGCGAGG-3′, carrying a BamHI restriction site (underlined) and reverse primer: 5′-GCGCAAGCTTCTACTTGTACAGCTCGTCCATGCC-3′, carrying a HindIII restriction site (underlined). The amplified mCherry sequence was cloned into pTurboGFP-B vector (Evrogen, catalog #FP513) to replace the TurboGFP reporter. Plasmids carrying mCherry were amplified and transfected into *E. coli* (BL21-DE3) to produce constitutively mCherry-expressing *E. coli* strain (*E. coli*-mCherry). *E. coli*-mCherry was used for phagocytosis and bacterial degradation assays.

### Phagocytosis – preparation of control and Stx2-KD cells

Control and Stx2-KD macrophage cells (10^6^ each) were seeded onto 22-mm glass coverslips (Corning, catalog #CLS284522) placed within 6-well culture plates (Tarsons, catalog #980010) containing 2 ml of 5 µg/ml puromycin (Sigma-Aldrich, catalog #P7255) mixed complete DMEM per well. Plates were kept at 37°C for overnight within a humidified CO_2_ (5%) incubator. Cells were washed twice (2 ml×2) with warm (37°C) complete DMEM, 2 h before the initiation of phagocytosis, and finally maintained in 2 ml of warm (37°C) complete DMEM within a CO_2_ incubator until used.

### Phagocytosis – preparation of opsonized phagocytic particles

Zymosan particles and latex beads were opsonized with human IgG (Sigma-Aldrich, catalog #I4506) 12 h before use. Unlabeled zymosan particles (Thermo Fisher Scientific, catalog #Z2849) and zymosan particles labeled with Alexa Fluor 594 (zymosan red; Thermo Fisher Scientific, catalog #Z23374) were suspended in 500 µl PBS and sonicated briefly to dissociate any aggregates. Particles were finally reconstituted in PBS (10^8^/ml) and opsonized overnight at 4°C by incubating with 1 mg/ml human IgG. Opsonized unlabeled zymosan particles and opsonized zymosan red particles are denoted as OPZ and OPZR, respectively. Zymosans labeled with pHrodo red (pHRZ; Thermo Fisher Scientific, catalog #P35364) were suspended in 500 µl PBS and vortexed to dissociate any clumped particles. Reconstituted pHRZ particles in PBS (10^8^/ml) were supplemented with 1 mg/ml human IgG and incubated overnight at 4°C for opsonization and named OpHRZ. Latex beads of 0.8 µm diameter (Sigma-Aldrich, catalog #L1398) and 3 µm diameter (Sigma-Aldrich, catalog #LB30) were washed three times with PBS by centrifugation (3000 ***g***, 5 min) and finally suspended in PBS (5×10^8^/ml) to opsonize with 1 mg/ml human IgG for overnight at 4°C. Opsonized beads of 0.8 µm and 3 µm diameters were named OPB-0.8 and OPB-3.0, respectively. After opsonization, zymosan particles and latex beads were washed three times (3000 ***g***, 5 min) with ice-cold PBS and stored at 4°C in complete DMEM. *E. coli-*mCherry were opsonized with anti-LPS antibody (Cloud-Clone Corp, catalog #MAB526Ge24) 1 h before use. *E. coli*-mCherry were put into culture in Luria broth the day before its usage and grown overnight at 37°C to reach mid-log phase (OD_600_ 0.5–1.0). 5 ml culture was washed twice in PBS (2000× ***g***, 5 min) and subjected to another round of low-speed centrifugation (300 ***g***, 5 min) to remove any bacterial clusters. 100 µl of bacterial suspension was then opsonized with 10 µl of anti-LPS antibody for 1 h in room temperature followed by two PBS washes (2000 ***g***, 5 min) and resuspension and storage at 4°C in complete DMEM. Opsonized *E. coli*-mCherry is abbreviated as OPEC. All opsonized particles were vortexed and quantified before use.

### Phagocytosis – macrophage challenge with opsonized particles

Phagocytosis experiments were performed according to published methodologies with minor adaptations (Czibener et al., 2006; Huang et al., 2009). Briefly, control or Stx2-KD RAW 264.7 macrophages (10^6^ each) were seeded overnight on 22-mm glass coverslips (Corning, catalog #CLS284522) placed within 6-well culture plates (Tarsons, catalog #980010). Cells were then challenged with OPZ, OPZR, OpHRZ or OPB-3 at 15:1 (particle:cell) ratio. OPB-0.8 (intended for phagosome purification) and OPEC (intended to assay bacterial uptake and degradation) were used at 50:1 (particle:cell) ratio. After addition of relevant opsonized particles, plates were immediately centrifuged at 1000 rpm for 1 min (Hermle Z366K fitted with 221.16 rotor) to settle the particles uniformly on the macrophages. Plates were then quickly placed in a warm (37°C) humidified CO_2_ (5%) incubator to initiate synchronized phagocytosis. Phagocytosis events were terminated at different time points (as indicated in the figure legends) by replacing the medium with ice-cold PBS. Particle engagement, phagocytic uptake and phagosome maturation were analyzed by fluorescence imaging, flow cytometry, phagosome fractionation and SEM, where applicable, as stated below.

### Phagocytosis – macrophage-engaged particle quantification

To compare engagement of opsonized particles between control and Stx2-KD macrophages, OPZR or OPEC challenged macrophages were washed thoroughly with ice-cold PBS at the indicated time points. Cells were then fixed with 2% paraformaldehyde (PFA) for 10 min followed by three PBS washes. Excess PBS was adsorbed by placing tissue papers at the edge of the coverslips. Coverslips were subsequently mounted on glass slides using a DAPI (nuclear stain)-containing mounting medium (Thermo Fisher Scientific, catalog #P36962). Images were acquired using a Leica SP8 confocal microscope with 63×/1.4 NA oil immersion objectives. Both DIC and fluorescent images were recorded choosing arbitrary fields. Cell-associated particles were counted manually and normalized to nuclear counts (DAPI positive). The extent of opsonized particle engagement in larger populations of cells was analyzed by flow cytometry.

### Phagocytosis – Phagocytosis index and phagocytosis rate determination

To analyze the phagocytic index and phagocytic rate, macrophage-engaged particles were first distinguished as internalized and non-internalized particles by differential inside-outside staining (Montaño et al., 2018). Opsonized particle-engaged macrophages were fixed with 2% PFA for 10 min, washed three times with PBS and blocked with 0.2% gelatin in PBS for 1 h, followed by 2 h incubation with Alexa Fluor 488-conjugated anti-human-IgG secondary antibodies (1:100 dilution; for OPZR-engaged cells) or Alexa Fluor 488-conjugated anti-mouse-IgG secondary antibodies (1:100 dilution; for OPEC engaged cells), diluted in 0.2% gelatin in PBS. Secondary antibodies cannot access completely internalized OPZR or OPEC, and thus can only bind to non-internalized particles to produce green–yellow fluorescence, whereas completely internalized particles produce only red fluorescence. The phagocytic index was calculated for each macrophage by multiplying the number of absolutely ingested particles/macrophage by 100, and is expressed as number (#) of ingested particles/100 macrophages (Seth et al., 2006). Phagocytic rate was estimated by first plotting number of completely engulfed particles/macrophage against time, followed by extrapolation of number of engulfed particles to a particular time point from linear fit curves.

### Phagocytosis – bacterial degradation assay

For bacterial degradation assay, macrophages grown on coverslips were exposed to OPEC for 2 h. Phagocytosis of OPEC was halted by ice-cold PBS wash. Macrophages were fixed and blocked as stated above, and external *E. coli* were labeled with Alexa Fluor 488-conjugated anti-mouse-IgG secondary antibodies (1:100 dilutions in 0.2% gelatin in PBS). Coverslips were mounted on glass slides and were imaged using a confocal fluorescence microscope to assess the number of engulfed bacteria (red only) and their morphology.

### Immunostaining and confocal microscopy

For immunostaining, macrophages grown overnight on coverslips were washed in PBS and fixed in 2% PFA in PBS for 10 min at room temperature (RT). Following three PBS washes (5 min each), cells were permeabilized with 0.1% Triton X-100 in PBS for 2 min, washed three times in PBS (5 min each) and subsequently blocked with 0.2% gelatin in PBS (blocking buffer) for 10 min at RT. Cells intended for surface staining were skipped for permeabilization and directly used for blocking after fixation and PBS wash. Primary antibodies diluted in blocking buffer were used for 1 h to immunostain cells for Stx2 (Synaptic Systems, catalog #110123, 1:200 dilution; R&D Systems, catalog #AF2568, 1:200 dilution), Rab5A (Cell Signaling Technology, catalog #46449, 1:100 dilution), Rab11 (Cell Signaling Technology, catalog #46449, 1:200 dilution), VAMP4 (Thermo Fisher Scientific, catalog #PA1-768, 1:100 dilution), VAMP7 (Thermo Fisher Scientific, catalog #PA5-116892, 1:100 dilution), LAMP1 (Thermo Fisher Scientific, catalog #14-1071-81, 1:500 dilution), Fcγ (Thermo Fisher Scientific, catalog #14-0161-82, 1: 100 dilution), TfR (Abcam, catalog #ab214039, 1:200 dilution), Cat L (R&D Systems, catalog #AF1515, 1:100 dilution), Cat B (Cell Signaling Technology, catalog #31718, 1:100 dilution), Cat D (Cloud-Clone Corporation, catalog #MAB280Hu22, 1:100 dilution) and Phalloidin647 (Thermo Fisher Scientific, catalog # A22287, 1:500 dilution). Cells were then washed three times in PBS (5 min each), followed by 1 h incubation with appropriate secondary antibodies (1:800 dilution) conjugated to Alexa Fluor 488, Alexa Fluor 594 or Alexa Fluor 647. After three PBS washes, coverslips were tapped gently on tissue papers to remove excess PBS and were mounted using a mounting medium containing nuclear stain DAPI (Thermo Fisher Scientific, catalog #P36962). A *Z*-series of confocal images were captured at a 0.34 µm interval with a Leica SP8 confocal platform equipped with a 63×/1.4 NA oil immersion objective (Zeiss) with appropriate laser excitation and emission filters. Images were deconvoluted using Leica Lightning software. Fluorescence signal were quantified using Zen (Carl Zeiss) software. Fluorescence intensity [in arbitrary units (A.U.)] for each data point in control cells was normalized by dividing by the average value. This normalization step transformed the control values into relative values centered on 1. Average value from the control was then used to normalize the raw intensity values (in A.U.) from the treatment groups by dividing each raw value by the average control value. The Pearson coefficient (*r*) for colocalization was measured using the ImageJ software. We considered colocalization significant for *r*≥0.5. The colocalization was considered moderate for r values 0.3 to <0.5. For r values that fall in between 0.1 to 0.3, we considered the colocalization insignificant (Dunn et al., 2011).

### Flow cytometry

All FACS analyses were performed by a BD LSR Fortessa flow cytometer using appropriate filter set up and analyzed with BD FACSDiva software. Lysotracker staining and FACS analyses were performed following a published methodology (Zhang et al., 2022) with minor adjustments. Control and Stx2-KD cells were trypsinized and brought into suspension (1.2×10^6^ cells/ml) in complete DMEM followed by 15 min incubation with 50 nM Lysotracker Red DND-99 at 37°C in a humidified CO_2_ (5%) incubator. Cells were then pelleted at 1000 ***g*** for 3 min, resuspended in 50 nM Lysotracker Red DND-99 containing ice-cold PBS and analyzed immediately. For surface staining of FcRs, 1.2×10^6^ cells were fixed in 1% PFA (in PBS) for 5 min followed by 0.2% gelatin blocking for 15 min at RT. The cells were incubated with rat anti-FcR (anti-CD16/CD32) antibody (1:200 dilution; Thermo Fisher Scientific, #14-0161-82; Czibener et al., 2006) for 1 h at 4°C, followed by three PBS washes (5 min×3) and subsequent incubation with anti-rat-IgG conjugated to Alexa Fluor 488 (1:800 dilution) for 1 h at 4°C. Cell were again washed three times with PBS (5 min×3) and finally resuspended in 1 ml PBS before FACS analyses. For the detection of phagocytic particle engagement, RAW 264.7 cells were washed with ice-cold PBS to halt uptake of fluorescent particles (OPZR and OPEC). Cells were then trypsinized and brought into suspension in ice-cold PBS and spun down at 300 ***g*** for 3 min at 4°C to remove aggregates. 0.8 ml of supernatants in ice-cold PBS containing 10^6^ cells/ml were subjected to FACS analyses.

### SEM

Monolayers of RAW 264.7 cells on glass coverslips were incubated with OPB-3 (1:15, cell:OPB) or OPEC (1:100, cell:bacteria) and arrested for phagocytosis at different times as elaborated above. Cells were then fixed in 2.5% glutaraldehyde (in PBS, pH 7.4) for 1 h at 4°C. After washing with PBS twice, cells were osmicated for 20 min in 1% (v/v) aqueous osmium tetroxide at RT. Cells were then washed with PBS, followed by dH_2_O and dehydrated by 5 min sequential incubations in 30, 50, 60, 80 and 100% ethanol. Samples were further dried overnight in a vacuum desiccator and imaged using a Zeiss Supra 55VP scanning electron microscope operating at 5.2 kV. Acquired SEM images were opened in ImageJ, and the scale was calibrated to ensure area calculation in µm^2^ and that the phagocytic cup region in each macrophage was manually outlined by a researcher doing who was aware of the experimental conditions using the freehand selection tool in ImageJ. Then the area of the selected region was measured in µm^2^ using the ‘measure’ command.

### TfR recycling assay

The TfR recycling assay was performed as previously described (Sheff et al., 2002), with some minor modifications. Briefly, three sets (Set-1, Set-2 and Set-3) of control and Stx2-KD macrophages (serum starved for 30 min) were incubated with 5 µg/ml of Alexa Fluor 568-conjugated transferrin (Tf-568, Thermo Fisher Scientific, catalog #T23365) for 5 min at room temperature (RT) to allow binding to surface TfR. Cells were then washed with ice-cold PBS to remove unbound Tf-568. Set-1 cells were fixed immediately with 2% PFA for the detection of surface TfR. Set-2 and Set-3 cells were shifted to 37°C in serum-free DMEM to allow internalization of Tf-568. Set-2 cells were taken out after 30 min, followed by washing and fixation to measure internalized TfR as well those still remaining on the surface. Set-3 macrophages were maintained at 37°C for another 30 min to allow surface recycling of internalized TfR (Baravalle et al., 2005) and were then incubated at RT with 5 µg/ml of Alexa Fluor 488-conjugated transferrin (Tf-488, Thermo Fisher Scientific, catalog #T13342) to label recycled TfR. Cells were then washed twice and fixed. Macrophages were then imaged using a confocal microscope to detect the distribution of Tf-568 and surface abundance of Tf-488.

### Purification of phagosomes

Phagosomes were purified as described previously with minor adjustments (Desjardins et al., 1994; Huang et al., 2009; Vinet and Descoteaux, 2009). Briefly, control or Stx2-KD cells were grown on 150 mm Petri dishes (Corning, catalog #CLS430599) to obtain ∼4×10^7^ cells for each condition. Cells were then incubated with OPB-0.8 at a ratio of 50:1 (beads: cell) for 30 min to 1 h. Subsequently, the cells were washed with warm DMEM to eliminate unbound OPB-0.8 and either used for purification immediately (for 30 min OPB-0.8 incubated cells to obtain nascent phagosomes) or allowed to mature for another 30 min (for 1 h OPB-0.8 incubated cells). For phagosome isolation, cells were then washed and scrapped in ice-cold PBS, and pelleted at 1200 ***g*** at 4°C. Pellets were washed with 10 ml ice-cold homogenization buffer [HB, 8.55% sucrose, 3 mM imidazole and 2× protease inhibitor cocktail (PIC; Sigma-Aldrich, catalog #P8340), pH 7.4] for 5 min and pelleted at 1200 ***g***. Pellets were brought to suspension in 1 ml HB with 2× PIC and passed through a 30-G needle (fitted in a 1 ml syringe) until >90% of cells were broken, as confirmed by phase microscopy. The homogenates were clarified at 1200 ***g*** for 5 min and supernatants enriched with OPB-0.8 containing phagosomes were mixed with an equal volume of 62% sucrose. This mixture was layered over 3 ml of 62% sucrose, followed by sequential topping with 2 ml each of 35, 25 and 10% sucrose (all prepared in 3 mM imidazole, pH 7.4) in SW40 tubes (344060, Beckman). The samples were then centrifuged at 24,100 rpm for 1 h at 4°C using a SW40Ti rotor (Beckman). Phagosomes were recovered from the interface of 10 and 25% sucrose (Fig. S6) using a pipette, washed with 12 ml ice-cold PBS, and harvested at 40,000 ***g***.

### Subcellular fractionation

∼90% confluent RAW 264.7 cells in 100 mm dishes were washed twice in PBS and scrapped into 200 µl of NP-40 lysis buffer (l0 mM Tris-HCl pH 7.9, 140 mM KCl, 5 mM MgCl_2_ and 0.5% NP-40) (Brady et al., 2018) supplemented with PIC and kept on ice for 15 min. Cells were passed through a 22 G syringe for five to ten times until ≤90% of cells were broken (verified under the light microscope). Lysates were then spun at 100 ***g*** for 5 min to pellet down unbroken cells. Supernatants were collected and further centrifuged at 1000 ***g*** for 5 min to pellet down nuclei. The resultant supernatant represents the cytosolic plus membrane fraction. The nuclear pellets were washed twice in NP-40 lysis buffer and then sonicated in Laemmli sample buffer equal to the volume of the cytosolic plus membrane fraction. Equal volume of nuclear and cytosolic plus membrane fractions were clarified by SDS-PAGE along with 20 µg of total cell lysates (TCL).

### Cathepsin secretion assay

An equal number (10^7^) of control and Stx2-KD RAW 264.7 macrophages were seeded on 100 mm tissue culture plates and allowed to adhere for 6 h. Macrophages were then washed three times with serum-free DMEM to remove any non-adherent cells and finally kept in 5 ml of serum-free DMEM for 12 h. Conditioned media were collected and subjected to centrifugation (1000 ***g***, 5 min) to remove any cells. 4 ml of each conditioned medium was then subjected to acetone (Sigma-Aldrich, catalog #179124) precipitation by mixing with 20 ml of pre-cooled (−20°C) acetone, followed by 2 h incubation at −20°C. Precipitated proteins were pelleted at 13,000 ***g*** for 20 min at 4°C. Pellets were air-dried to remove traces of acetone and finally dissolved in 200 µl of Laemmli buffer. 20 µl of each sample were clarified by SDS-PAGE along with 20 µg of TCL. The presence of cathepsins in secretion was detected by western blotting.

### Western blotting

RAW 264.7 cells or purified phagosomes were lysed in ice-cold RIPA buffer (Santa Cruz Biotechnology; catalog #sc-24948) with a protease inhibitor cocktail (PIC, 1×; Sigma-Aldrich, catalog #P8340). Samples were then sonicated (Sartorius) and separated from insoluble debris at 10,000 ***g*** for 10 min at 4°C. Protein concentration was estimated using modified Lauri method and brought to similar level by adding RIPA buffer containing PIC. Lysates were then boiled in Laemmli buffer (1.5×), resolved by 10–12% SDS–PAGE and transferred to nitrocellulose membranes using a semi-dry transfer system (Bio-Rad). Non-specific binding sites were blocked in 5% skim milk dissolved in TBST [TBS (50 mM Tris-HCl pH 7.4, 274 mM NaCl, 9 mM KCl) with 0.05% Tween-20] for 1 h at RT and membranes were washed briefly to remove excess milk. Primary antibodies diluted in primary antibody buffer [1% BSA (w/v) in 50 mM Tris-HCl pH 7.4)] were used to probe membranes for Stx2 (Thermo Fisher Scientific, catalog #PA5-87903, 1:1000 dilution), Rab5A (Cell Signaling Technology, catalog #46449; 1:1000 dilution), VAMP4 (Thermo Fisher Scientific, catalog #PA1-768, 1:1000 dilution), LAMP1 (R&D Systems, catalog #AF4320, 1:1000 dilution), Golgin160 (Santa Cruz Biotechnology, catalog #sc-374596, 1:500 dilution), VAMP7 (Thermo Fisher Scientific, catalog #PA5-116892, 1:1000 dilution), VAMP8 (Proteintech, catalog #15546-1-AP, 1:1000 dilution), cathepsin L (R&D Systems, catalog #1515, 1:1000 dilution), cathepsin B (Cell Signaling Technology, catalog #31718, 1:1000 dilution), cathepsin D (Proteintech, catalog #21327-1-AP, 1:1000 dilution), ATP6V1A (Cell Signaling Technology, catalog #39517, 1:1000 dilution), TFEB (Thermo Fisher Scientific, catalog #PA5-96632, 1:1000 dilution), histone H3 (Cell Signaling Technology, catalog #9715, 1:2000 dilution), LC3A/B (Cell Signaling Technology, catalog #12741, 1:400 dilution), SQSTM1/p62 (Cell Signaling Technology, catalog #39749, 1:800 dilution), β-actin (Cell Signaling Technology, catalog #3700, 1:1000 dilution) and tubulin (Cell Signaling Technology, catalog #3873; 1:1000 dilution) for 12 h at 4°C. After washing three times (10 min×3) with TBST, membranes were incubated with suitable HRP-conjugated secondary antibodies diluted (1: 5000) in 5% skim milk in TBST for 1 h at RT. Membranes were then washed three times in TBST (10 min×3), developed with ECL reagent and documented using a ChemiDoc imaging system (Bio-Rad). Membranes containing phagosome samples were stained with Ponceau S (5 min) after semi-dry transfer to document loading and used to wash with TBST to remove stain before proceeding for blocking. Densitometry analyses of the western blot bands were performed using ImageJ 1.54i software (NIH, USA). Band density values from control samples were normalized to 1 and the band density values from treated samples were divided by 1 to generate relative density values. Uncropped western blot images are provided in Fig. S8 with references to their respective main figures.

### Statistical analysis

Two-tailed unpaired Student's *t*-test statistical analyses were performed using GraphPad Prism software (v.9.3.0). All the values are expressed as means±s.e.m. All experiments were repeated independently for at least three times. The actual numbers of experiments and cells or phagosomes quantified are mentioned in the individual figure legends.

## Supplementary Material

10.1242/joces.263855_sup1 Supplementary information
