# Supplementary material for: Syntaxin-2 balances phagocytic uptake and phagolysosomal clearance in macrophages
Source: J Cell Sci. 2025 Aug 6;138(15):jcs263855. doi: 10.1242/jcs.263855 (PMC12377716; doi:10.1242/jcs.263855)
Supplement: Supplementary information [file joces-138-263855-s1.pdf]

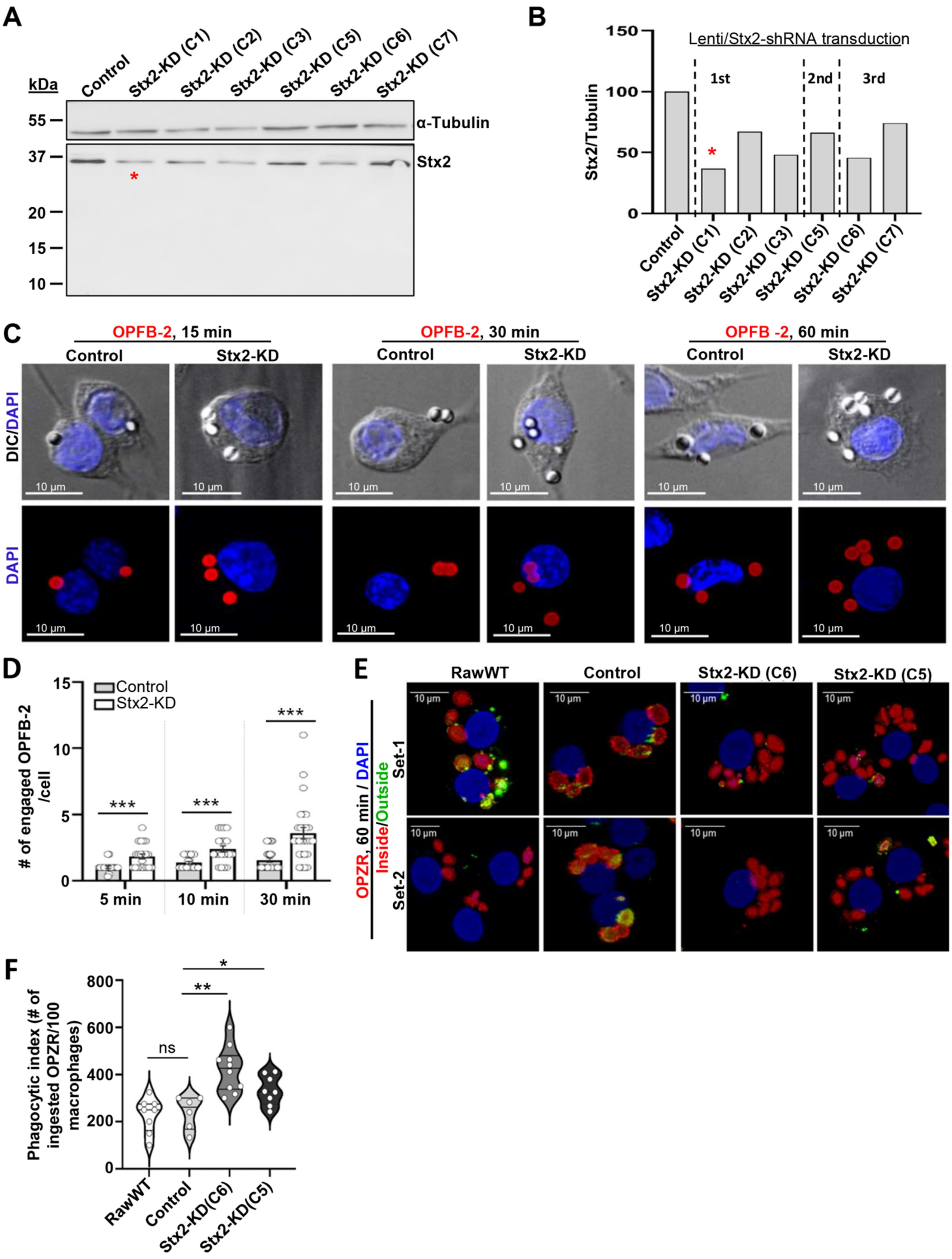

Journal of Cell Science • Supplementary information

Fig. S1. Lenti/Stx2-shRNAs significantly deplete endogenous Stx2 in RAW264.7 macrophages and increase engagement and uptake of IgG-opsonized particles. (A) Representative western blots from Lenti/sc-shRNA transduced (Control) or Lenti/Stx2-shRNA transduced (Stx2-KD) RAW 264.7 cells to test Stx2 depletion. C1-C3 and C5-C7 represents the parent puromycin resistant colonies obtained from 3 independent transduction experiments. Tubulin used as loading control. (B) Quantification of Stx2 band densities normalized to tubulin. "*" indicates the colony (C1 from 1st transduction) with maximum Stx2 depletion (Stx2-KD: ~70%) that further amplified and used in all the experiments stated in this article. Macrophages from C5 (Stx2-KD: ~35%, from 2nd transduction) and C6 (Stx2-KD: ~60%, from 3rd transduction) colonies were used to validate fundamental observations on C1. (C) Representative DIC (upper panel) and fluorescence images (bottom panel) created by stacking 3 consecutive optical sections from Control or Stx2-KD macrophages challenged with IgG-opsonized fluorescent beads of 2 µm diameter (OPFB-2) for 15, 30 and 60 min. DAPI (blue) shows the nuclei. Scale bars, 10 µm. (D) Quantification of the macrophage engaged OPFB-2 in Control and Stx2-KD macrophages (15min Control, n=20; Stx2-KD, n= 21), (30min Control, n=25; Stx2-KD, n= 20), (60min Control, n=25 ; Stx2-KD, n= 27) N = 3 independent experiments. Results are mean±s.e.m. ***P < 0.001. (E) Maximum intensity projection images of untreated macrophages (WT), Control macrophages and macrophages from C5 and C6 clones, incubated with OPZR for 60 min followed by inside/outside staining to differentiate completely (red) and partially ingested (green/yellow) OPZR. Scale bars, 10 µm. (F) Quantification of phagocytic index for OPZR from E (WT, n= 60; Control, n= 55; C6, n= 110; C5, n= 80). N = 3 independent experiments. Results shown as violin plots with median and quartiles marked. ns = not significant, *p < 0.05, **p < 0.01. All statistical tests were two-tailed unpaired Student's t-tests.

Journal of Cell Science • Supplementary information

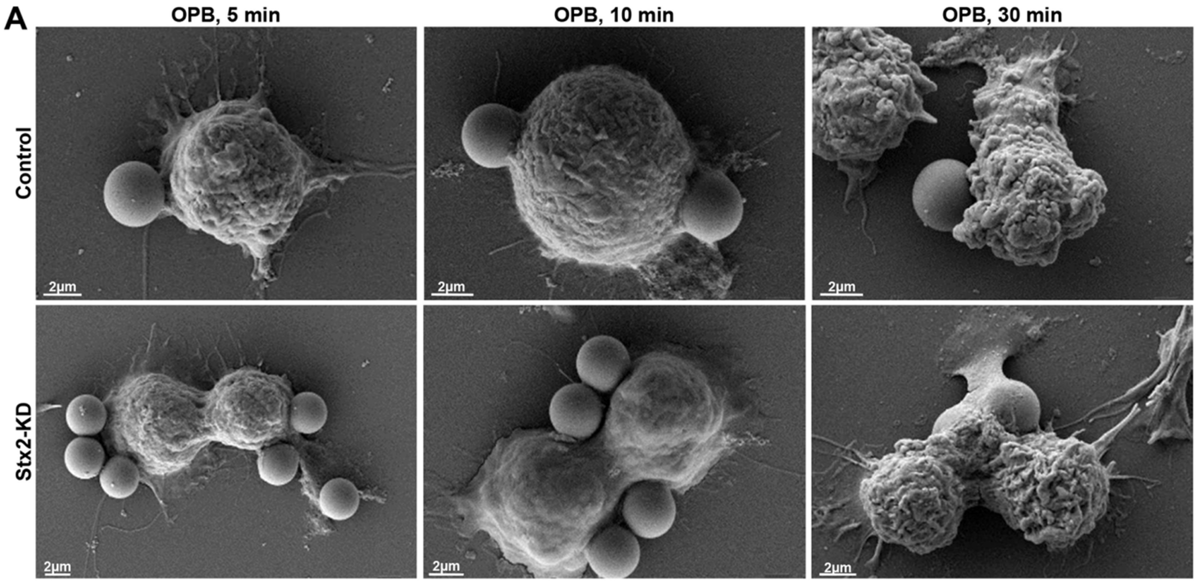

Fig. S2. Increased OPB-3 engagement on Stx2-KD macrophage surface. (A) Additional SEM images of Control (upper panel) and Stx2-KD (bottom panel) macrophages incubated with OPB-3 for the indicated time. Stx2-KD cells are showing more efficiency for OPB attachment.

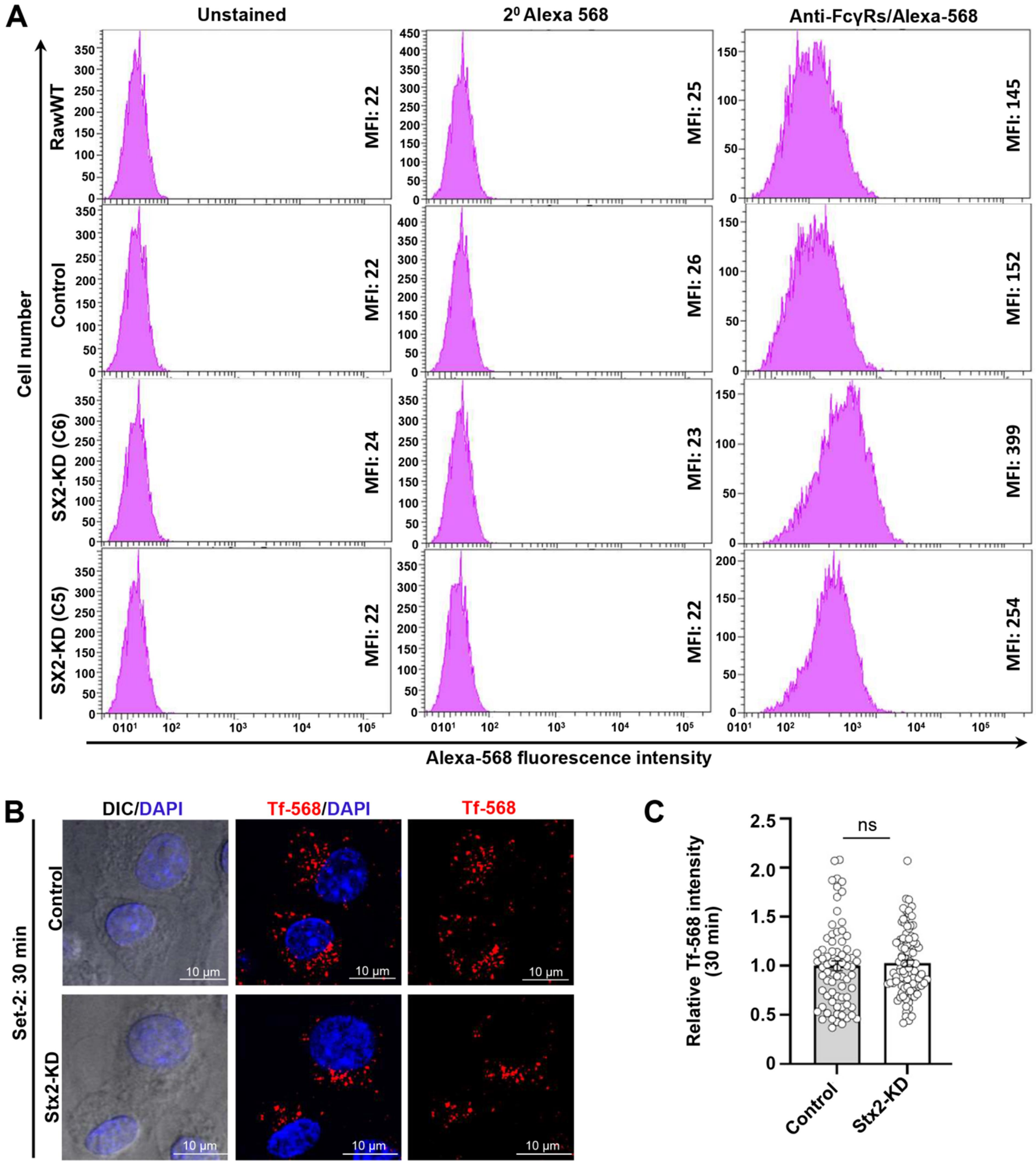

Fig. S3. Stx2 depletion augments surface-expressed FcR in macrophages. (A) FACS histograms with mean fluorescence intensity (MFI) of unlabeled, only 2^0-antibody labeled and FcR labeled WT, Control and C6 and C5 macrophages. (B) Distribution of Tf-568 labeled TfR in Set-2 macrophages, 30 min after activation of endocytosis. (C) Quantification of Tf-568 fluorescence intensity in macrophages from Set-2. Control, n= 74 and Stx2-KD, n= 98. N = 3 independent experiments. Results are mean±s.e.m. ns = not significant. Stx2-KD macrophages show equivalent intensity for Tf-568, despite increased initial surface labeling (Set-1, Fig. 3K,L) aligns with elevated recycling that resulted further decrease after 60 min (Set-3; Fig. 3K, right panel; Fig. 3L, right panel). All statistical tests were two-tailed unpaired Student's t-tests.

Journal of Cell Science • Supplementary information

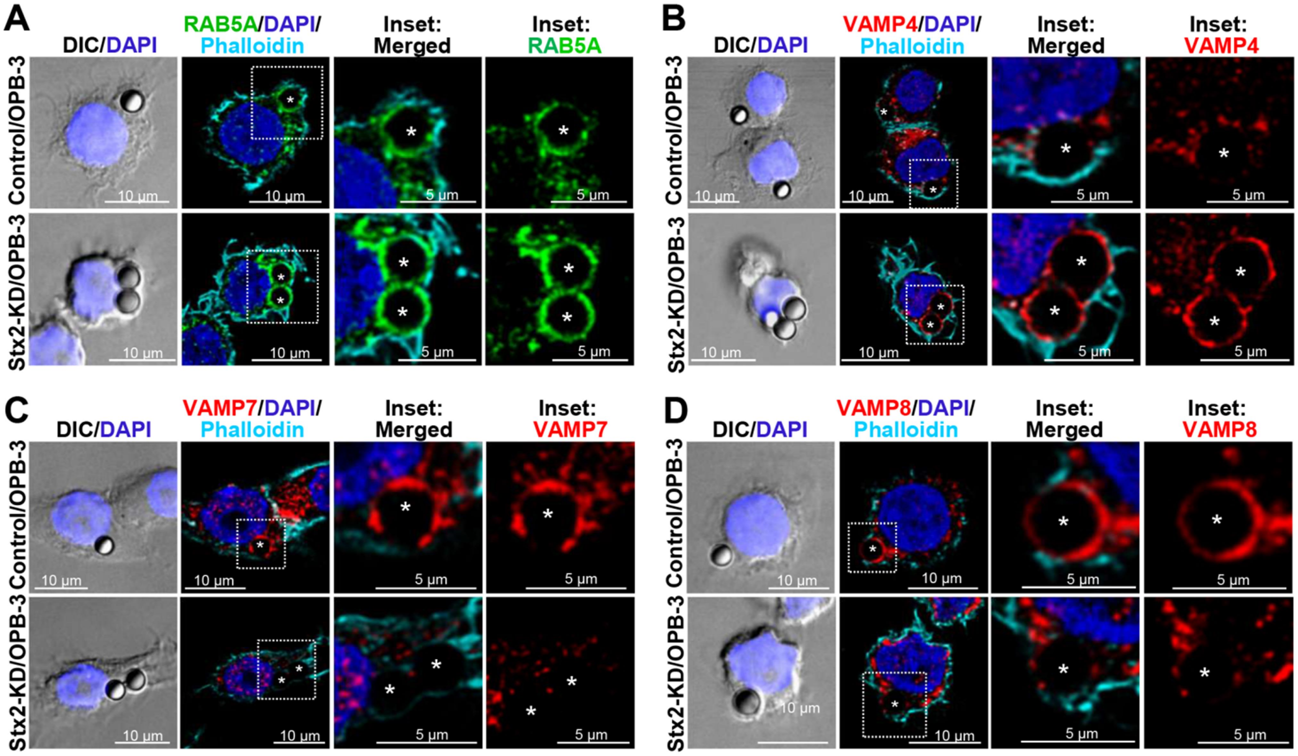

Fig. S4. Stx2 depleted nascent phagosomes possess more of early endosomes and VAMP4-positive compartments. (A-D) DIC and confocal images of 30 min OPB-3 challenged macrophages probed for F-actin (phalloidin) and (A) Rab5A, (B) VAMP4, (C) VAMP7 and (D) VAMP8. DAPI shows the nuclei. White stars mark the OPB-3 containing phagosomes. Boxed regions enlarged in the insets. Scale bars 10 and 5 µm (inset).

Journal of Cell Science • Supplementary information

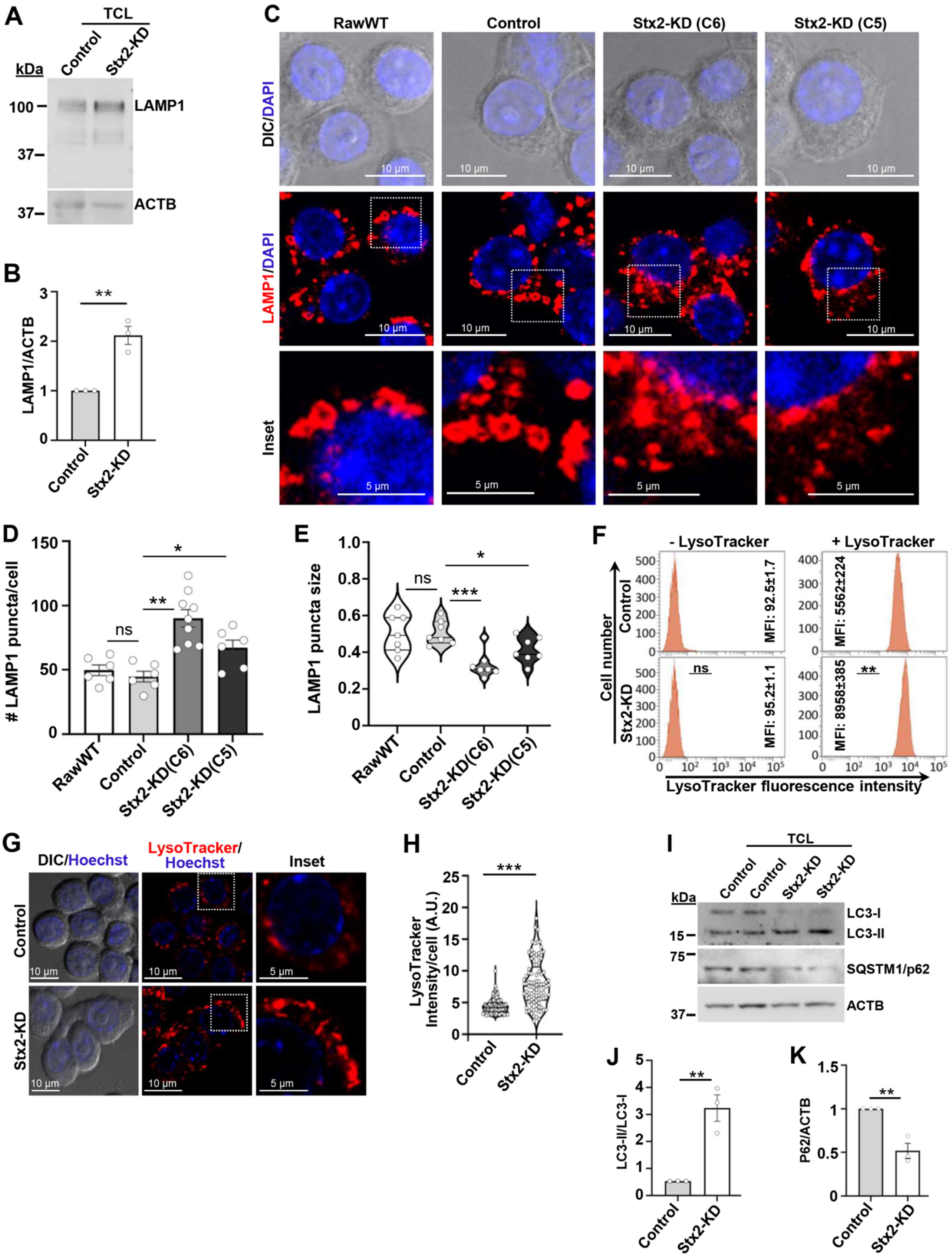

Journal of Cell Science • Supplementary information

Fig. S5. Stx2 depletion in macrophage augments the content of acidic and functional lysosomes: (A) Representative western blots show expression of LAMP1 and loading control ACTB in total cell lysates (TCL) from Control and Stx2-KD macrophages. (B) Quantification of LAMP1 band density normalized to ACTB loading control. N = 3 independent experiments. Results are mean±s.e.m.. **P < 0.01. (C) DIC and confocal images of WT, Control, C5 and C6 macrophages, probed for LAMP1. Boxed regions are magnified in inset. Nucleus (blue) stained with DAPI. Scale bars, 10 µm, 5 µm (inset). (D, E) Quantification of (D) the number and (E) size of LAMP1 puncta in WT, Control, C5, and C6 macrophages. At least 30 cells (n) were quantified for each condition. N = 3 independent experiments. For D, results are mean±s.e.m. E shown as violin plots with median and quartiles marked. ns = not significant, *P < 0.05, **P < 0.01, ***P < 0.001. (F) FACS histograms and the mean MFI values (as mean±s.e.m) of 3 histograms from LysoTracker stained RAW 264.7 macrophages. ns = not significant, **P < 0.01. (G) Representative confocal DIC and fluorescence images of Control and Stx2-KD macrophages stained with LysoTracker Red DND-99. Boxed regions are magnified in inset. Nucleus (blue) stained with Hoechst. Scale bars, 10 µm, 5 µm (inset). (H) Quantification of LysoTracker Red intensity (in arbitrary unit: A.U.) from individual Control and Stx2-KD macrophages (Control, n= 93; Stx2-KD, n= 118). N = 3 independent experiments. Results shown as violin plots with median and quartiles marked. ***P < 0.001. (I) Representative western blots of total cell lysates (TCL) from Control and Stx2-KD macrophages probed for LC3-I and II, SQSTM1/p62 and loading control ACTB. (J,K) Quantification of band density ratio for (J) LC3-II to LC3I and (K) SQSTM1 (p62) normalized to ACTB. N = 3 independent experiments. Results are mean±s.e.m.. **P < 0.01. All statistical tests were two-tailed unpaired Student's t-tests.

Journal of Cell Science • Supplementary information

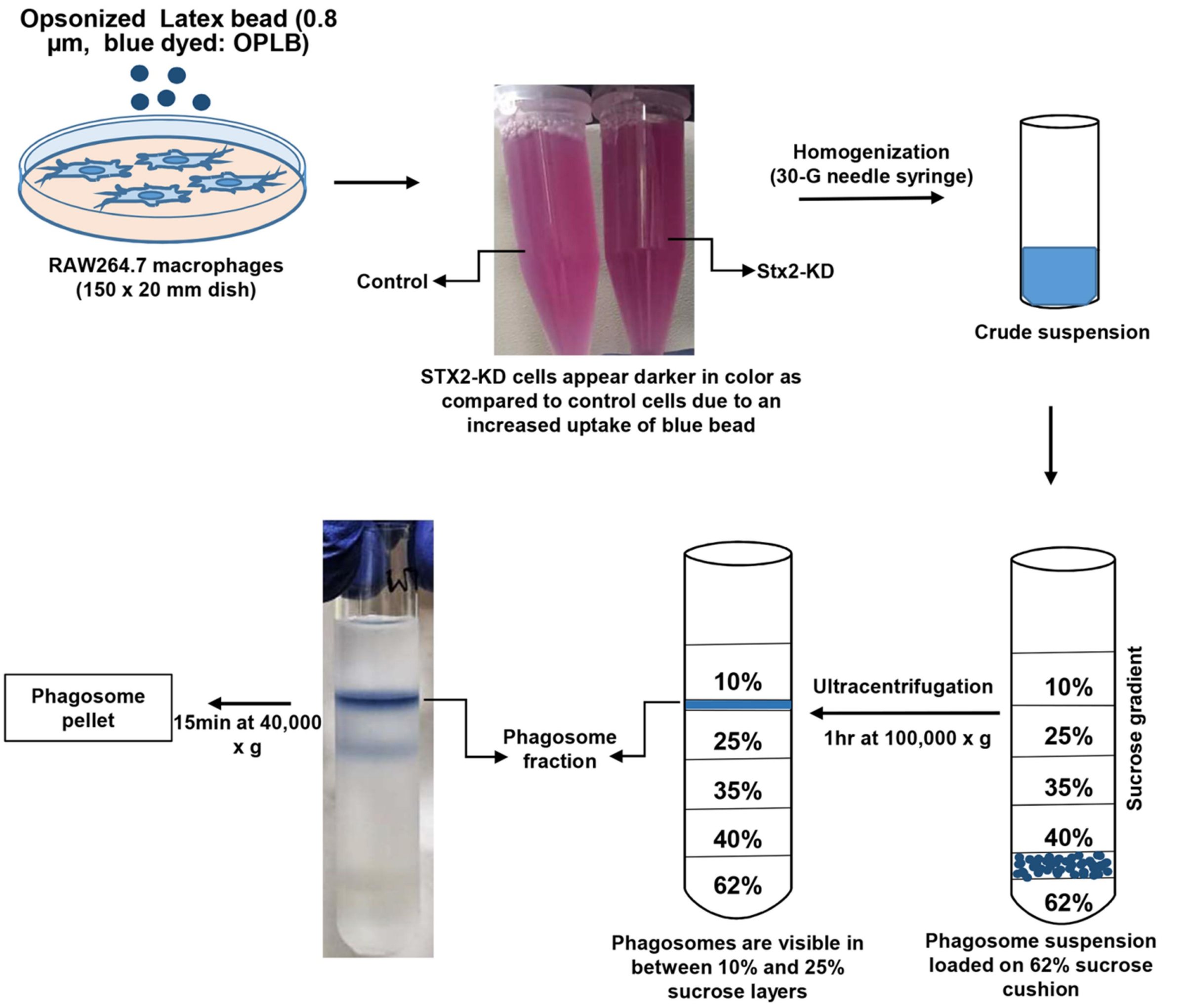

Fig. S6. Schematic presentation of the phagosome purification methodology, described in detail in the "materials and method" section. OPB-0.8 containing phagosomes were purified by using sucrose density gradient centrifugation as outlined.

Journal of Cell Science • Supplementary information

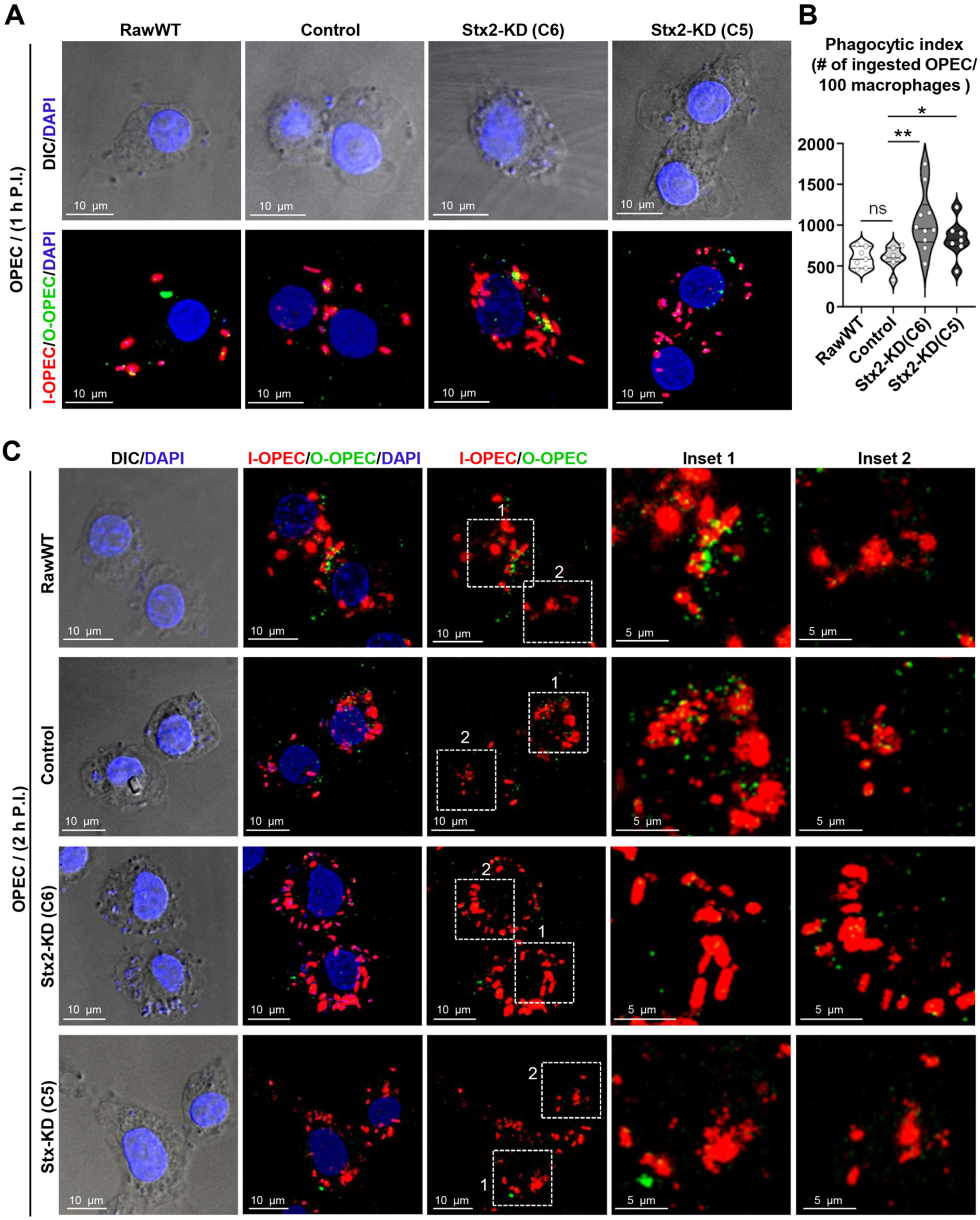

Journal of Cell Science • Supplementary information

Fig. S7. Stx2 depleted macrophages from independently generated C5 and C6 clones display uncontrolled uptake and increased bacterial load for IgG-opsonized *E. coli* (OPEC) akin to C1 macrophages. (A) Maximum intensity projection confocal images of WT, Control, C6 and C5 macrophages incubated for 1 h with OPEC and subjected to inside (red)/outside (green) staining. (B) C6 and C5 clones showed increased phagocytic index for OPEC, while phagocytic index of WT and Control macrophages remained same. At least n = 45 cells were analyzed. N = 3 independent experiments. Results shown as violin plots with median and quartiles marked. ns = not significant, *P < 0.05, ***P < 0.001. (C) Maximum intensity projection confocal images of WT, Control, C6 and C5 macrophages incubated for 2 h (1 h uptake + 1 h maturation) with OPEC and subjected to. Inside (red)/outside (green) staining. WT and Control macrophages were able to degrade OPEC and displayed low bacterial load, while OPEC remained relatively intact and showed higher bacterial load in C6 and C5 macrophages. All statistical tests were two-tailed unpaired Student's t-tests.

A (Correspond to **Fig. 1H**)

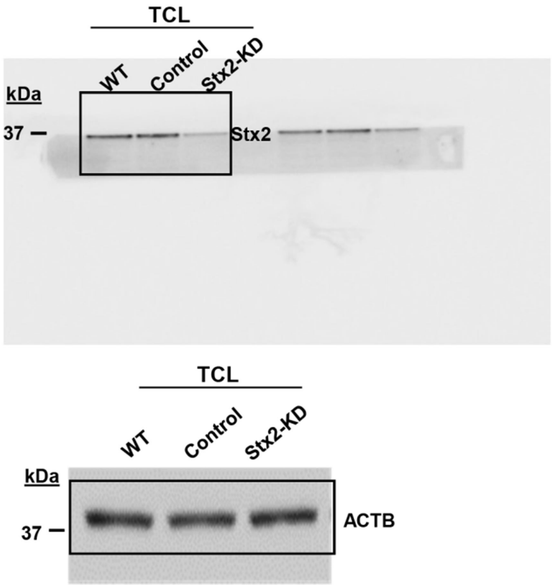

B (Correspond to **Fig. S1A**)

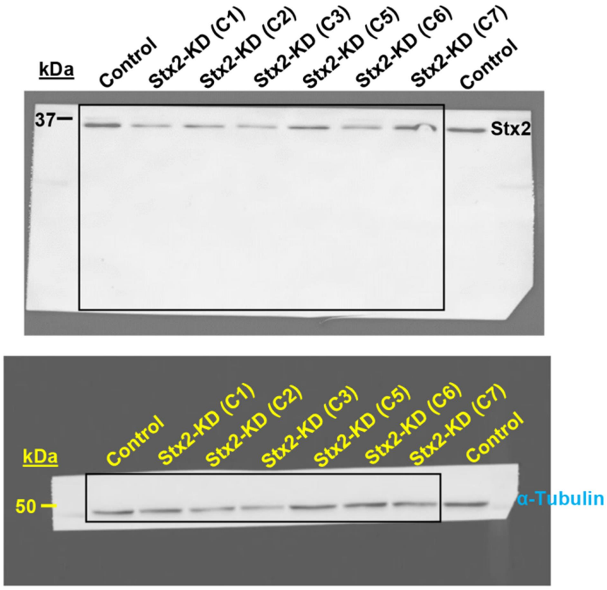

C (Correspond to **Fig. 3I**)

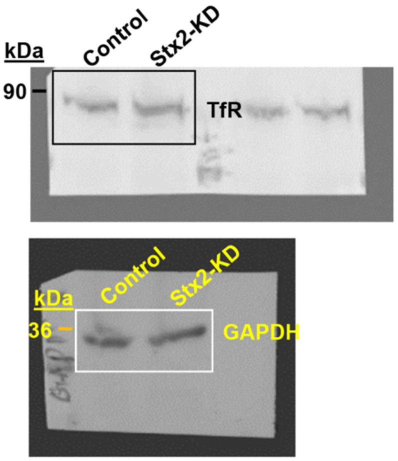

D (Correspond to **Fig. 4E**)

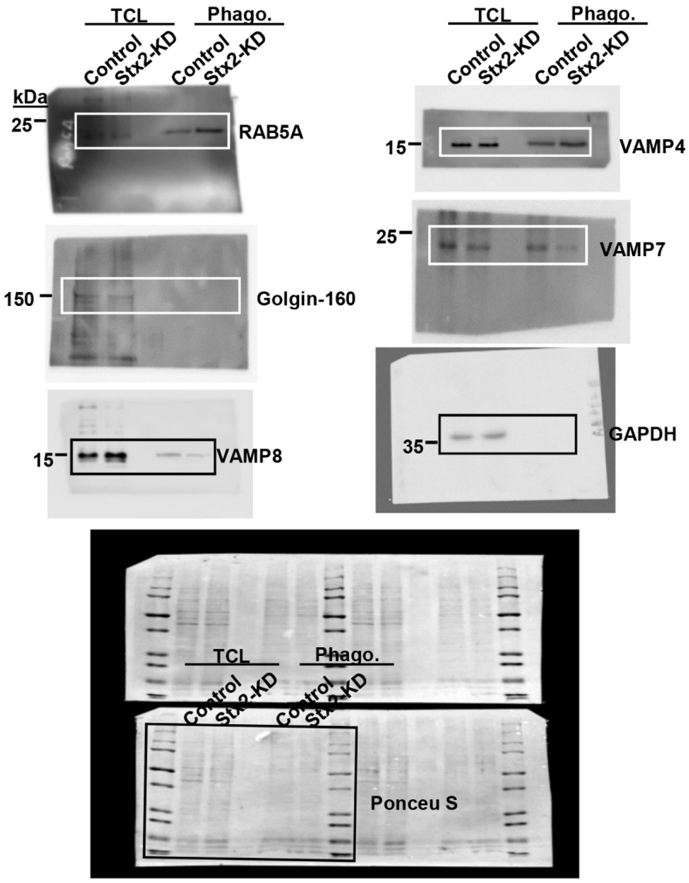

Journal of Cell Science • Supplementary information

E (Correspond to **Fig. 5E)**

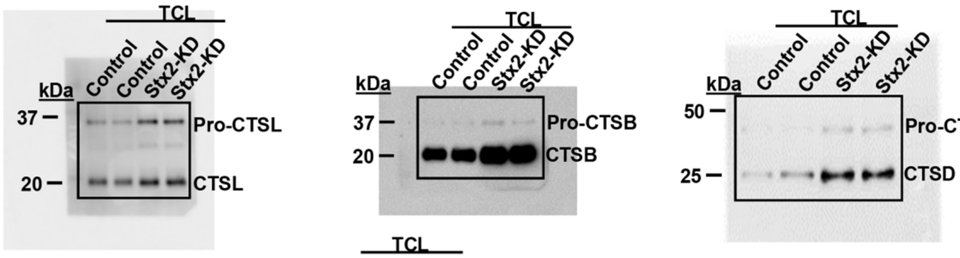

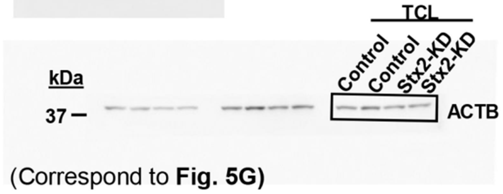

G (Correspond to **Fig. 5I)**

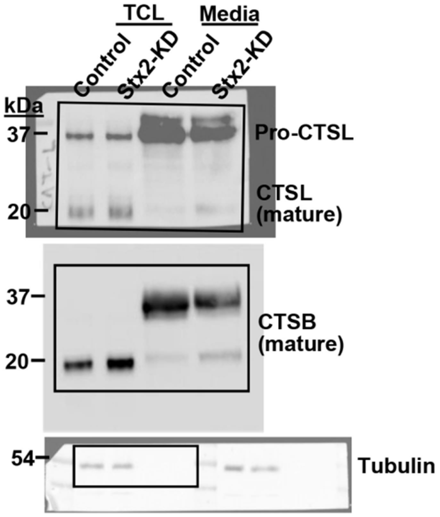

F (Correspond to **Fig. 5G)**

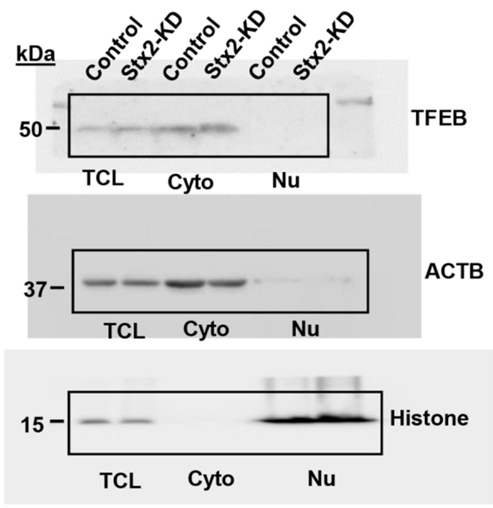

H (Correspond to **Fig. S5A)**

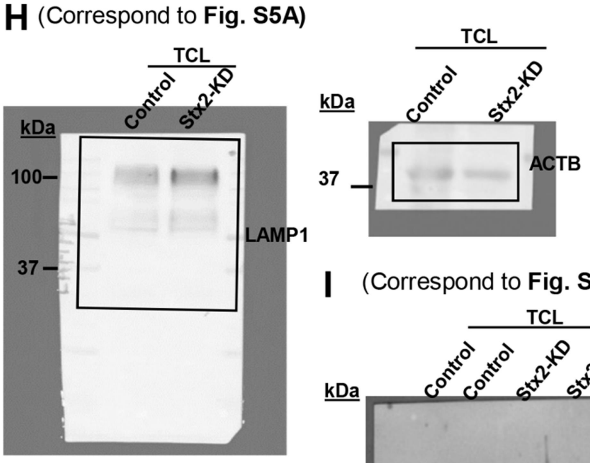

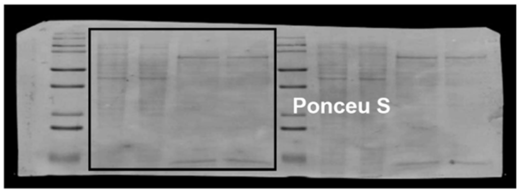

I (Correspond to **Fig. S5F)**

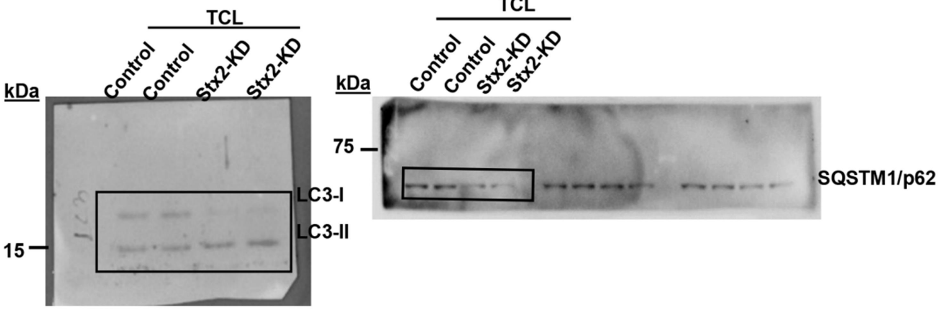

J (Correspond to **Fig. 6E**)

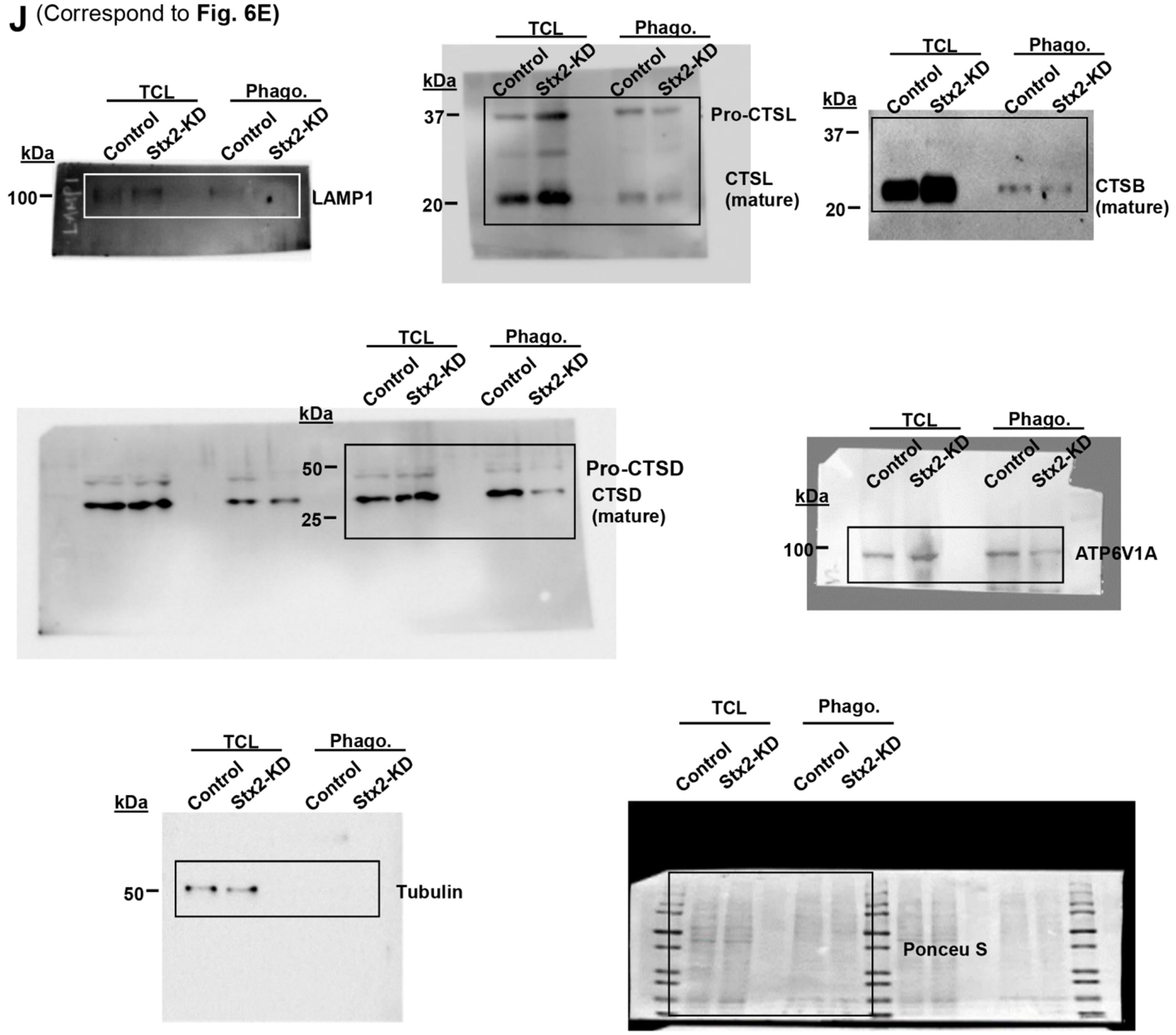

Fig. S8. Original western blot images for blot transparency. (A-J) Uncropped western blot images used in this manuscript with boxes highlighting the regions used for cropping. The blots are displayed in the same order as they appear in the main figures, and their corresponding main figure numbers are indicated alongside the labeling.

Journal of Cell Science • Supplementary information

Table S1. List of antibodies used for this study.

Antibody	Source	Identifier
**Primary antibodies**		
Anti-ATP6V1A (E5N9E), Rabbit monoclonal	Cell Signaling Technology (Danvers, MA, USA)	Cat# 39517; RRID:AB_3083783
Anti-β-Actin, Mouse monoclonal	Cell Signaling Technology (Danvers, MA, USA)	Cat# 3700; RRID:AB_2242334
Anti-Cathepsin B (CTSB), Rabbit monoclonal	Cell Signaling Technology (Danvers, MA, USA)	Cat# 31718; RRID:AB_2687580
Anti-Cathepsin D (CTSD), Rabbit polyclonal	Proteintech (Rosemont, IL, USA)	Cat# 21327-1-AP; RRID:AB_10733646
Anti-Cathepsin D (CTSD), Mouse monoclonal	Cloud-Clone Corporation (Katy, TX, USA)	Cat# MAB280Hu22, RRID: Not available
Anti-Cathepsin L (CTSL), Goat polyclonal	R&D Systems (Minneapolis, MN, USA)	Cat# AF1515; RRID:AB_2087690
Anti-CD16/CD32 (Fcγ), Rat Monoclonal	Thermo Fisher Scientific (Waltham, MA, USA)	Cat# 14-0161-82; RRID:AB_467133
Anti-GAPDH, Rabbit polyclonal	Bio Bharati Life Science (Kolkata, WB, India)	Cat# BB-AB0060 RRID: Not available
Anti-Golgin 160, Mouse monoclonal	Santa Cruz Biotechnology (Santa Cruz, CA, USA)	Cat# sc-374596; RRID:AB_10989547
Anti-Histone H3 Antibody, Rabbit polyclonal	Cell Signaling Technology (Danvers, MA, USA)	Cat# 9715; RRID:AB_331563
Anti-LAMP1/CD107a, Rat monoclonal	Thermo Fisher Scientific (Waltham, MA, USA)	Cat# 14-1071-81; RRID:AB_657532
Anti-LAMP1/CD107a, Goat monoclonal	R&D Systems (Minneapolis, MN, USA)	Cat# AF4320; RRID:AB_2296826
Anti-LC3A/B, Rabbit monoclonal	Cell Signaling Technology (Danvers, MA, USA)	Cat# 12741; RRID:AB_2617131
Anti-Lipopolysaccharide (LPS), Mouse monoclonal	Cloud-Clone Corporation (Katy, TX, USA)	Cat# MAB526Ge24; RRID: Not available
Anti-SQSTM1/p62, Rabbit	Cell Signaling Technology	Cat# 39749;

monoclonal	(Danvers, MA, USA)	RRID:AB_2799160
Anti-Rab5A, Mouse monoclonal	Cell Signaling Technology (Danvers, MA, USA)	Cat# 46449; RRID:AB_2799303
Anti-Syntaxin-2, Rabbit polyclonal	Synaptic Systems (Göttingen, Germany)	Cat# 110123; RRID:AB_887849
Anti-Syntaxin-2, Rabbit polyclonal	Thermo Fisher Scientific (Waltham, MA, USA)	Cat# PA5-87903; RRID:AB_2804493
Anti-Syntaxin-2, Mouse polyclonal	R&D Systems (Minneapolis, MN, USA)	Cat# AF2568; RRID:AB_2302866
Anti-TFEB, Rabbit polyclonal	Thermo Fisher Scientific (Waltham, MA, USA)	Cat# PA5-96632; RRID:AB_2808434
Anti-Transferrin Receptor, Rabbit monoclonal	Abcam (Cambridge, UK)	Cat# ab214039, RRID:AB_2904534
Anti-Tubulin, Rabbit polyclonal	Cell Signaling Technology (Danvers, MA, USA)	Cat# 3873; RRID:AB_1904178
Anti-VAMP4, Rabbit polyclonal	Thermo Fisher Scientific (Waltham, MA, USA)	Cat# PA1-768; RRID:AB_2212790
Anti-VAMP7, Rabbit polyclonal	Thermo Fisher Scientific (Waltham, MA, USA)	Cat# PA5-116892; RRID:AB_2901522
Anti-VAMP8, Rabbit polyclonal	Proteintech (Rosemont, IL, USA)	Cat# 15546-1-AP; RRID:AB_2878150
IgG from human serum	Sigma-Aldrich (St Louis, MO, USA)	Cat# I4506; RRID:AB_1163606
**Secondary antibodies**		
Anti-goat Donkey IgG, Alexa Fluor 594-conjugated	Jackson ImmunoResearch (West Grove, PA, USA)	Cat# 705-585-003; RRID:AB_156820
Anti-goat Rabbit IgG, HRP conjugate	Millipore (Burlington, MA, USA)	Cat# AP106P; RRID:AB_92411
Anti-human Goat IgG, Alexa Fluor 488-conjugated	Thermo Fisher Scientific (Waltham, MA, USA)	Cat# A-11013; RRID:AB_141360
Anti-mouse Donkey IgG, Alexa Fluor 488-conjugated	Jackson ImmunoResearch (West Grove, PA, USA)	Cat# 715-545-150; RRID:AB_167505

Journal of Cell Science • Supplementary information

Anti-mouse Goat IgG, Alexa Fluor 488-conjugated	Thermo Fisher Scientific (Waltham, MA, USA)	Cat# A-11004; RRID:AB_2534072
Anti-mouse Rabbit IgG, HRP-conjugated	Millipore (Burlington, MA, USA)	Cat# AP160P; RRID:AB_92531
Anti-rabbit Donkey IgG, Alexa Fluor 488-conjugated	Jackson ImmunoResearch (West Grove, PA, USA)	Cat# 711-545-152; RRID:AB_168465
Anti-rabbit Goat IgG, Alexa Fluor 594-conjugated	Jackson ImmunoResearch (West Grove, PA, USA)	Cat# 111-585-003; RRID:AB_163359
Anti-rabbit Goat IgG, Alexa Fluor Plus 647-conjugated	Thermo Fisher Scientific (Waltham, MA, USA)	Cat# A32733; RRID:AB_2633282
Anti-rabbit Goat IgG, HRP-conjugated	Thermo Fisher Scientific (Waltham, MA, USA)	Cat# 31460; RRID:AB_228341
Anti-rabbit Goat IgG, Alexa Fluor 488-conjugated	Thermo Fisher Scientific (Waltham, MA, USA)	Cat# A-11034; RRID:AB_2576217
Anti-rat Goat IgG, Alexa Fluor 488-conjugated	Thermo Fisher Scientific (Waltham, MA, USA)	Cat# A-11006; RRID:AB_2534074
Anti-rat Goat IgG, Alexa Fluor 568-conjugated	Thermo Fisher Scientific (Waltham, MA, USA)	Cat# A-11077; RRID:AB_2534121

Table S2. List of reagents used for this study.

Reagent	Source	Identifier
Acetone	Sigma-Aldrich (St Louis, MO, USA)	Cat# 179124
Bovine Serum Albumin	Sigma-Aldrich (St Louis, MO, USA)	Cat# A2058
DMEM	Thermo Fisher Scientific (Waltham, MA, USA)	Cat# 12100061
DMSO, Anhydrous	Thermo Fisher Scientific (Waltham, MA, USA)	Cat# D12345
Fetal Bovine Serum (FBS)	Avantor (Radnor, PA, USA)	Cat# 97068-085
Gelatin	Amresco (Dallas, TX, USA)	Cat# 9764

Journal of Cell Science • Supplementary information

Glutaraldehyde solution	Sigma-Aldrich (St Louis, MO, USA)	Cat# G7776
Hoechst 34580	Thermo Fisher Scientific (Waltham, MA, USA)	Cat# H21486
Laemmli Sample Buffer (2X)	Bio-Rad (Hercules, CA, USA)	Cat# 1610737
Latex beads, deep blue dyed 0.80 µm average diameter	Sigma-Aldrich (St Louis, MO, USA)	Cat# L1398
Latex beads, polystyrene 3.0 µm mean particle size	Sigma-Aldrich (St Louis, MO, USA)	Cat# LB30
L-Glutamine	Thermo Fisher Scientific (Waltham, MA, USA)	Cat# 25030081
LysoTracker™ Red DND-99	Thermo Fisher Scientific (Waltham, MA, USA)	Cat# L7528
Methanol	Hi-Media (Thane, Maharashtra, India)	Cat# MB113
Nitrocellulose membrane, 0.2 µm	Bio-Rad (Hercules, CA, USA)	Cat# 1620112
Paraformaldehyde	Sigma-Aldrich (St Louis, MO, USA)	Cat# P6148
Penicillin-Streptomycin (10,000 U/mL)	Thermo Fisher Scientific (Waltham, MA, USA)	Cat# 15140122
Phalloidin, Alexa Fluor™ 647 conjugated	Thermo Fisher Scientific (Waltham, MA, USA)	Cat# A22287
Phenylmethylsulfonyl fluoride (PMSF)	Sigma-Aldrich (St Louis, MO, USA)	Cat# 52332
Phosphate buffered saline, pH 7.4	Himedia (Thane, Maharashtra, India)	Cat# TS1101
Polybrene Infection / Transfection Reagent	Sigma-Aldrich (St Louis, MO, USA)	Cat# TR-1003
Ponceau S Staining Solution	Thermo Fisher Scientific (Waltham, MA, USA)	Cat# A40000279
ProLong™ Diamond Antifade Mountant with DAPI	Thermo Fisher Scientific (Waltham, MA, USA)	Cat# P36962

Journal of Cell Science • Supplementary information

Prestained Protein Standards	Bio-Rad (Hercules, CA, USA)	Cat# 1610377
Protease inhibitor cocktail	Sigma-Aldrich (St Louis, MO, USA)	Cat# P8340
Puromycin dihydrochloride	Sigma-Aldrich (St Louis, MO, USA)	Cat# P7255
RIPA Lysis Buffer System	Santa Cruz Biotechnology (Santa Cruz, CA, USA)	Cat# sc-24948
Skim Milk Powder	Sigma-Aldrich (St Louis, MO, USA)	Cat# 70166
SuperSignal™ West Pico PLUS Chemiluminescent Substrate	Thermo Fisher Scientific (Waltham, MA, USA)	Cat# 34577
Syntaxin 2 shRNA (m) Lentiviral Particles	Santa Cruz Biotechnology (Santa Cruz, CA, USA)	Cat# sc-41327-V
Control shRNA Lentiviral Particles-A	Santa Cruz Biotechnology (Santa Cruz, CA, USA)	Cat# sc-108080
Transferrin from Human Serum, Alexa Fluor™ 568 Conjugate	Thermo Fisher Scientific (Waltham, MA, USA)	Cat# T23365
Transferrin from Human Serum, Alexa Fluor™ 488 Conjugate	Thermo Fisher Scientific (Waltham, MA, USA)	Cat# T13342
Triton X-100	Sigma-Aldrich (St Louis, MO, USA)	X-100
Trypsin-EDTA (0.25%), phenol red	Thermo Fisher Scientific (Waltham, MA, USA)	Cat# 25200056
Zymosan A, unlabeled	Thermo Fisher Scientific (Waltham, MA, USA)	Cat# Z2849
Zymosan A, Alexa Fluor™ 594 conjugated	Thermo Fisher Scientific (Waltham, MA, USA)	Cat# Z23374
Zymosan BioParticles, pHrodo™ Red conjugated	Thermo Fisher Scientific (Waltham, MA, USA)	Cat# P35364

Journal of Cell Science • Supplementary information

Table S3. List of plasmids and oligonucleotides used for this study.

Plasmids/Oligonucleotides	Source	Identifier
pTurboGFP-B	Evrogen (Moscow, Russia)	Cat# FP513
mCherry-TFR-20	Addgene (Watertown, MA, USA)	Cat# 55144
mCherry forward: 5’ GCGCGGATCCATGGTGAGCAAG GGCGAGG 3’	This work	N/A
mCherry reverse: 5’ GCGCAAGCTTCTACTTGTACAGC TCGTCCATGCC 3’	This work	N/A

Journal of Cell Science • Supplementary information
